# Comparative effectiveness of different probiotics supplements for triple helicobacter pylori eradication: a network meta-analysis

**DOI:** 10.3389/fcimb.2023.1120789

**Published:** 2023-05-15

**Authors:** Yue Wang, Xue Wang, Xue-Yan Cao, Han-Long Zhu, Lin Miao

**Affiliations:** ^1^ Medical Centre for Digestive Diseases, the Second Affiliated Hospital of Nanjing Medical University, Nanjing, Jiangsu, China; ^2^ Department of Gastroenterology and Hepatology, Jinling Hospital, Affiliated Hospital of Medical School, Nanjing University, Nanjing, Jiangsu, China

**Keywords:** helicobacter pylori, treatment, probiotics, efficacy and safety, network meta-analysis

## Abstract

**Background:**

Probiotics has been reported as an effective supplement for Helicobacter pylori eradication. However, knowledge of their comparative efficacy is still lacking.

**Aim:**

In this study, we used network meta-analysis of current probiotics supplement used in standard triple therapy to assess and rank their comparative effectiveness.

**Methods:**

All randomized controlled trials from three main databases (PubMed, Embase and Cochrane Library) up to April 2022 were collected and filtered to meet our criterion. We used Bayesian network meta-analysis to evaluate the eligible randomized controlled trials and gave a rank for the efficiency and incidence of side effects of each probiotics supplement. The ranking probability for each therapy was assessed by means of surfaces under cumulative ranking values. Subgroup analysis was conducted to evaluate other possible influencing factors.

**Results:**

34 eligible randomized controlled trials entered the following meta-analysis, including 9,004 patients randomized to 10 kinds of therapies. Result showed that most probiotics added therapies had better outcomes than triple therapy, among which Bifidobacterium-Lactobacillus and Bifidobacterium-Lactobacillus-Saccharomyces adjuvant therapy could obtain comprehensive benefit with high eradication rate (78.3% and 88.2% respectively), and cause few side effects. Combination of different probiotics, adding probiotics before or after triple therapy and longer duration of probiotics can improve therapeutic effect in *H.pylori* infected individuals.

**Conclusion:**

For triple therapy of *H.pylori* infection, adding probiotics can increase eradication rate and bring protective effect. Considering the overall influence, Bifidobacterium-Lactobacillus or Bifidobacterium-Lactobacillus-Saccharomyces therapy can be a better choice in improving *H.pylori* eradication process.

## 1 Introduction

Helicobacter pylori (*H.pylori*) has been proven to be a human carcinogen, causing chronic gastric inflammation, which may lead to precancerous lesions of atrophic gastritis and intestinal metaplasia (Crowe, 2019). This deterioration has a close relationship with the increasing risk of gastric cancer and its severity and extent (Yao and Smolka, 2019). The huge burden of *H.pylori* infection poses a great problem to most regions in the world, with approximately 4.4 billion patients infected with *H.pylori* worldwide in 2015 (Hooi et al., 2017). As recommended by International consensus, *H.pylori* should be eradicated as long as it is diagnosed (Malfertheiner et al., 2017). Eradication of *H.pylori* has been reported to reduce gastric cancer risk among individuals in high-risk areas (Lee et al., 2016). Currently, triple therapy is recommended as first-line treatment by the guideline, which contains proton-pump inhibitors (PPI), 2 kinds of antibiotics (usually clarithromycin and amoxicillin/metronidazole), given for 7-14 days (Malfertheiner et al., 2017). However, antibiotics resistance prevalence has been a challenging problem due to the frequent use of these drugs. Furthermore, the side effects of antibiotics, such as diarrhea, nausea, and vomiting, reduce the compliance of patients to some extent, thus leading to a reduction in *H.pylori* eradication rates (Liu et al., 2018).

Probiotics is an emerging supplementation in *H.pylori* treatment (Chey et al., 2017; Mu et al., 2018; Chen et al., 2021), which refers to beneficial microorganisms living in human intestinal tract, which can regulate the balance of intestinal flora and exert the functions of body regulation, collective defense, disease prevention and treatment (Dore et al., 2019). Common probiotics used in clinical treatment includes Lactobacillus, Bifidobacteria, Streptococcus, Saccharomyces and other mixed preparation. Several studies have shown that probiotics can effectively relieve the clinical symptoms of patients with H.pylori infection, improving the curative effect of *H.pylori*, and reduce the incidence of adverse drug reactions (Chey et al., 2017; Song et al., 2018; Ji and Yang, 2020). However, the effect of probiotics on eradication of *H.pylori* is still under investigation. Previous meta-analysis compared the effects of combining probiotics, placebo, and standard therapy, and the result showed that a 14-day course of triple therapy plus probiotics neutralized the adverse effects of diarrhea and nausea, but did not improve the eradication of *H.pylori*, as compared to placebo(Lu et al., 2016). Network meta-analysis (NWM) is evidence evaluating tool for comparison of randomized controlled trials (RCT) with multiple interventions directly and indirectly. This study conducted network meta-analysis to evaluate the comparative effectiveness and their adverse effect of current probiotics supplements added to triple therapy.

## 2 Methods

### 2.1 Search strategy and data extraction

We searched PubMed, Embase, Cochrane Central Register of Controlled Trials for all the RCTs written in English until April 2022. The search words and/or Medical Subject Heading (MeSH) terms used for search are listed in **Table S1**. This meta-analysis was performed using the Preferred Reporting Items for Systematic Reviews and Meta-Analysis (PRISMA) statement. The Grading of Recommendations Assessment, Development and Evaluation (GRADE) was used to evaluate the quality of evidence from pairwise and NWM (Page et al., 2021) (**Table S2**).

### 2.2 Study selection and quality assessment

Studies meeting the following conditions were eligible in our network meta-analysis: (a) Randomized controlled trials; (b) Participants received eradication outcomes evaluation after at least 4 weeks of the therapy; (c) Participants received outcomes evaluation at least 4 weeks after the end of eradication; (d) At least 2 groups, including control (triple therapy with none or placebo) and experimental (triple therapy with at least one kind of probiotics supplement or a mixture) group, are compared in the studies; (e) Data in the study could be extracted; (f) Article written in English. In our NWM, the primary efficacy end point was *H.pylori* eradication rate, and secondary outcome was adverse effect. Exclusion criteria were as follows: (a) Final eradication rate was unknown; (b) Inappropriate randomization trail method; (c) No description of withdrawals and dropout; (d) Case reports, clinical guidance, comments, letters or reviews. In case of duplicate studies, or outcomes from the same study individuals, the latest or more complete one was selected into further analysis. Risk of bias and the strength evaluation of evidence were assessed based on the guideline of the Cochrane Collaboration (Higgins et al., 2011). The quality of evidence of each study was divided into three levels: low risk, high risk, and unclear risk. Two independent reviewers appraised the risk of bias and quality assessment of all studies, and any discrepancies were solved *via* discussion to reach the consensus.

### 2.3 Statistical analysis

Data was processed by using Review Manager (Version 5.4), Stata (Version 13.0), Addis (Version 1.16.5) and R (Version 4.2.1). The sample size was analyzed with intention-to-treat (ITT). To get a more conservative estimate of the 95% confidence intervals (CI), this study used a random-effects model (REM) to analyze the data for these results. P < 0.05 reflected the presence of significance, and an I^2^ statistic >50% indicted the heterogeneity (DerSimonian, 1996). What’s more, inconsistency was appraised, which is essential for conducting an NWM. Comparison-adjusted funnel diagrams were used to evaluate the influence of the small-scale trial results *via* checking the symmetry (Higgins et al., 2003). The surfaces under cumulative ranking (SUCRA) values were calculated to assess the cumulative ranking probability of each intervention method compared with an ideal method. SUCRA = 1 or 100% represents the best efficacy. For both *H.pylori* eradication rates and side effect, subgroup analyses were conducted based on the following factors: region, publication year, antibiotics type and duration, PPI type, follow-up time, the adding time and duration of probiotics.

## 3 Results

### 3.1 Study characteristics and quality evaluation

**Figure 1:**
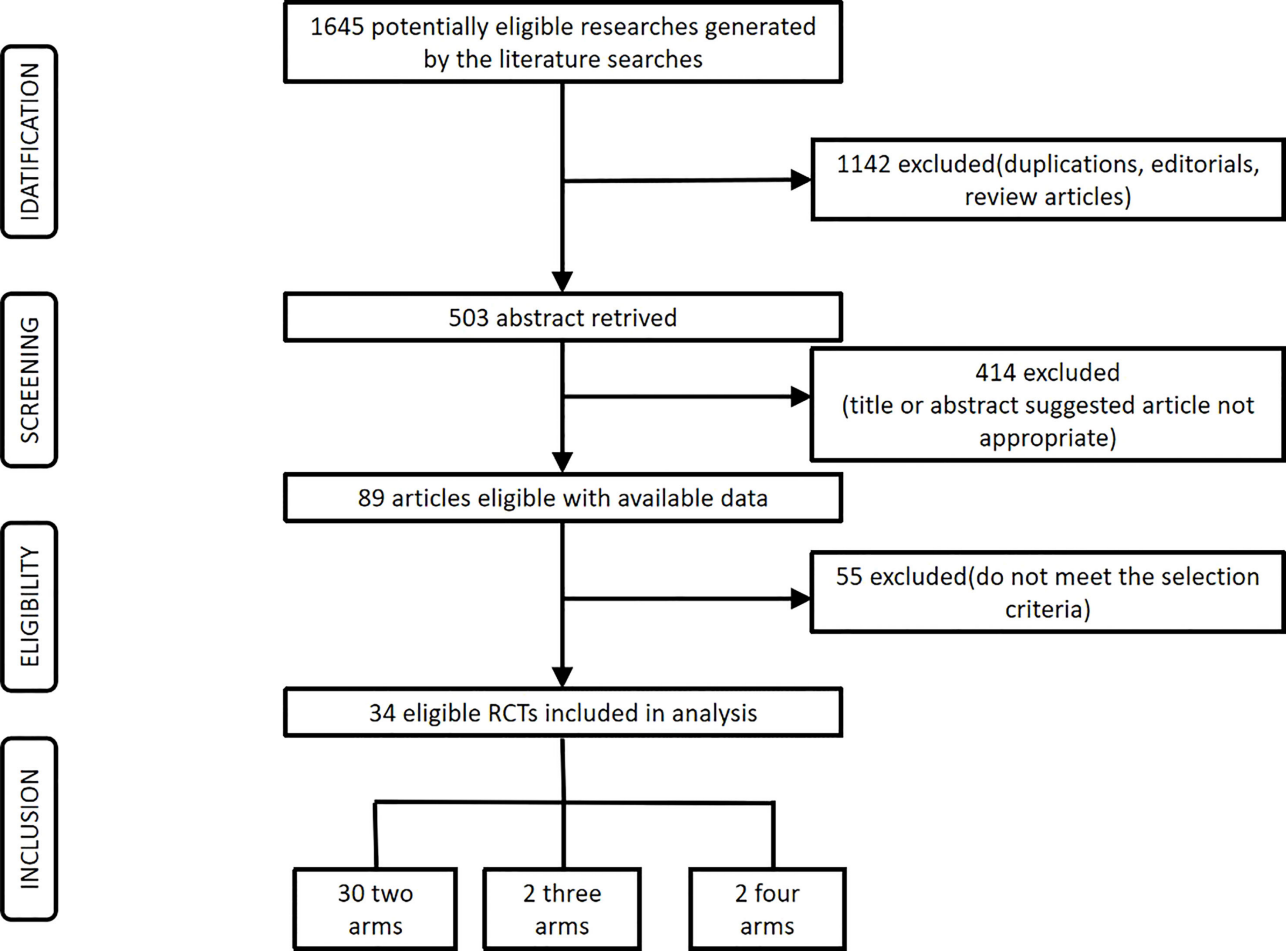
Flowchart of the study selection.

A total of 1,645 records were generated by the literature searches. Among these records, 1,142 articles were duplicate articles, or not RCT. 414 irrelevant articles were excluded after reading their titles or abstracts, and 55 articles were also be excluded because they did not meet our criteria. Finally, 34 potential eligible articles were further retrieved based on the selection criteria. The process was shown in **Figure 1**.

The 34 RCTs covered 10 kinds of interventions:

1. Triple therapy
2. Triple therapy with Bacillus
3. Triple therapy with Lactobacillus
4. Triple therapy with Saccharomyces
5. Triple therapy with Bifidobacterium-Lactobacillus
6. Triple therapy with Bacillus-Streptococcus
7. Triple therapy with Lactobacillus-Streptococcus
8. Triple therapy with Lactobacillus-Propionibacterium
9. Triple therapy with Bifidobacterium-Lactobacillus-Streptococcus
10. Triple therapy with Bifidobacterium-Lactobacillus-Saccharomyces

1 study (Jin and Kim, 2019) was a five-arm trail, 3 studies (Cremonini et al., 2002; Scaccianoce et al., 2008; Dajani et al., 2013) were four-arm trials, 6 studies (Ziemniak, 2006; Song et al., 2010; Ozdil et al., 2011; Du et al., 2012; D’Angelo et al., 2014; Chang et al., 2020) were three-arm trials and the remaining 24 studies were two-arm trials (Canducci et al., 2000; Armuzzi et al., 2001; Sheu et al., 2002; Nista et al., 2004; Duman et al., 2005; Myllyluoma et al., 2005; Shimbo et al., 2005; Sung et al., 2007; Lee et al., 2011; Medeiros et al., 2011; Yoon et al., 2011; Deguchi et al., 2012; Ojetti et al., 2012; Francavilla, 2013; Zojaji et al., 2013; Emara and Abdel-Aziz, 2014; Goran Hauser et al., 2014; Paoluzi et al., 2015; Tongtawee et al., 2015; Grgov et al., 2016; Haghdoost et al., 2017; Mihai, 2019; Muresan et al., 2019; Yang et al., 2021). The sample size of the trials ranged from 35 to 1500, containing totally 9004 participants, which were grouped into 40 paired comparisons/intervention arms. The baseline characteristics of the involved researches are listed in **Table S3**. The full articles of 7 RCTs were not available and the information was captured from their abstracts (Sung et al., 2007; Lee et al., 2011; Ozdil et al., 2011; Du et al., 2012; Goran Hauser et al., 2014; D’Angelo et al., 2014; Jin and Kim, 2019).

In terms of quality evaluation, the Cochrane Collaborations tool was used to assess the risk of bias. **Figures S1, S2** shows that 13 trials (Armuzzi et al., 2001; Duman et al., 2005; Sung et al., 2007; Lee et al., 2011; Ozdil et al., 2011; Ojetti et al., 2012; Dajani et al., 2013; D’Angelo et al., 2014; Tongtawee et al., 2015; Grgov et al., 2016; Haghdoost et al., 2017; Muresan et al., 2019; Chang et al., 2020) were judged as at high risk of bias, 14 trials (Nista et al., 2004; Myllyluoma et al., 2005; Shimbo et al., 2005; Ziemniak, 2006; Scaccianoce et al., 2008; Medeiros et al., 2011; Yoon et al., 2011; Deguchi et al., 2012; Du et al., 2012; Zojaji et al., 2013; Emara and Abdel-Aziz, 2014; Paoluzi et al., 2015; Mihai, 2019) as moderate, and the remaining 7 trials (Canducci et al., 2000; Cremonini et al., 2002; Sheu et al., 2002; Song et al., 2010; Goran Hauser et al., 2014; Jin and Kim, 2019; Yang et al., 2021) as low of bias. The bias almost came from the lack of allocation concealment or blinding to the treatment arms.

### 3.2 Network map

**Figure 2:**
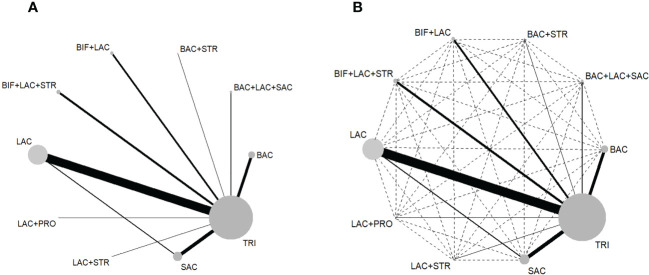
**(A)** Network map of the 10 direct comparisons included in all the RCTs. **(B)** Network map of all 50 comparisons in this NWM, including 10 direct (solid lines) and 40 indirect (interrupted lines). BAC, Bacillus; BIF, Bifidobacterium; LAC, Lactobacillus; PRO, Propionibacterium; SAC, Saccharomyces; STR, Streptococcus; TRI, triple therapy.

The NWMs comparing eradication rate (**Figure 2**) and side effects (**Figure S3**) of regimens show all the possible comparisons, direct evidence existed in eradication rate comparison for 10 pairs (**Figure 2A**) and 8 pairs in side effect comparison (**Figure S3A**), while indirect comparisons had 40 pairs (**Figure 2B**) and 28 pairs (**Figure S3B**) in eradication and side effect respectively. Node size represents the sample size of each treatment, while the width of edges is weighted according to the inverse of the variance in logarithm of the relative risk.

### 3.3 Network meta-analysis

#### 3.3.1 Direct and indirect pair comparisons, publication bias

The overall results estimating *H.pylori* eradication and weight of each RCT are shown in **Figure S4**. The overall RR value was 1.14(95% CI, 1.07, 1.21). The forest plot of **Figure S7** shows the RRs (95% CIs) of all direct pair comparisons grouped in 10 regimens pairwise eradication rate meta-analysis.

Among the comparisons related to eradication rate, triple therapy with Lactobacillus vs triple therapy (RR, 0.92; 95% CI, 0.87-0.97), triple therapy with Bacillus-Streptococcus vs triple therapy (RR, 0.86; 95% CI, 0.77-0.97) and triple therapy with Bifidobacterium-Lactobacillus-Saccharomyces vs triple therapy (RR, 0.88; 95% CI, 0.78-0.99) yielded significant results. In contrast, other pairs yielded insignificant results.

Tests of inconsistency suggested that insignificant overall results (P= 0.46) between direct and indirect measures, and the variance parameter was similar between random effects standard deviation (Median, 0.35; 95% CI, 0.11-0.62) and inconsistency standard deviation (Median, 0.36; 95% CI, 0.01-1.13). No significant publication bias was observed in the relevant funnel plot, which appears symmetrical (**Figures S5**, **S6**).

**Figure 3:**
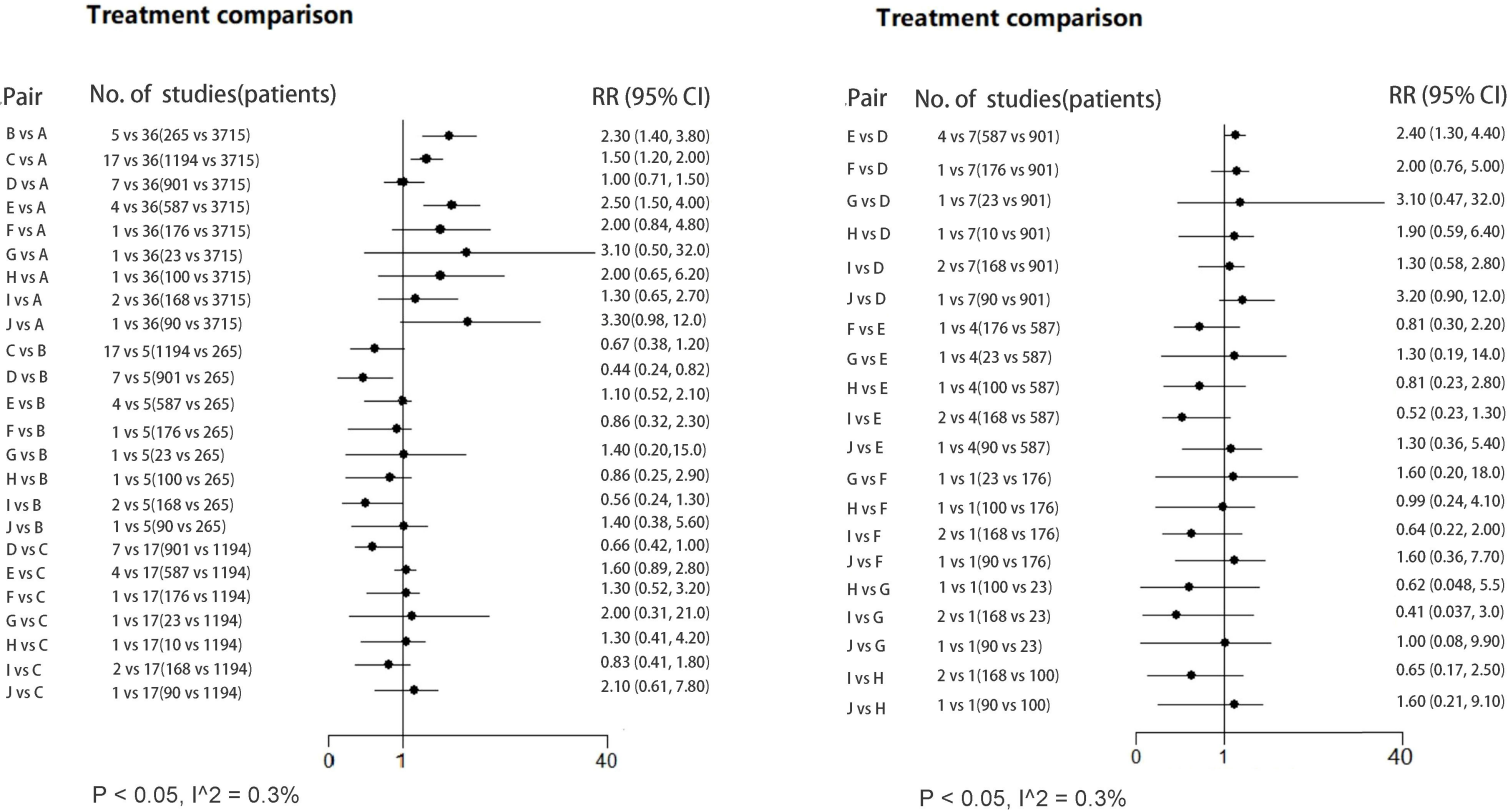
Forest plot (RR; 95% CI) illustrating all direct/indirect pair comparisons of regimens included in all the RCTs. RR, risk ratio; Regimen labels: A: triple therapy; B: triple therapy with Bacillus; C: triple therapy with Lactobacillus; D: triple therapy with Saccharomyces; E: triple therapy with Bifidobacterium+Lactobacillus; F: triple therapy with Bacillus+Streptococcus; G: triple therapy with Lactobacillus+Propionibacterium; H: triple therapy with Lactobacillus+Streptococcus; I: triple therapy with Bifidobacterium+Lactobacillus+Streptococcus; J: triple therapy with Bifidobacterium+Lactobacillus+Saccharomyces.

The network forest plot (**Figure 3**) shows RRs (95% credible intervals, CI) of all direct and indirect comparisons in this NWM. Of these comparators, triple therapy with Bacillus, Lactobacillus and Bifidobacterium-Lactobacillus had significantly better effect than triple therapy, while other kinds of therapies failed to reach significance.

#### 3.3.2 League matrix, rank of grams, and surfaces under cumulative ranking values

**Figure 4:**
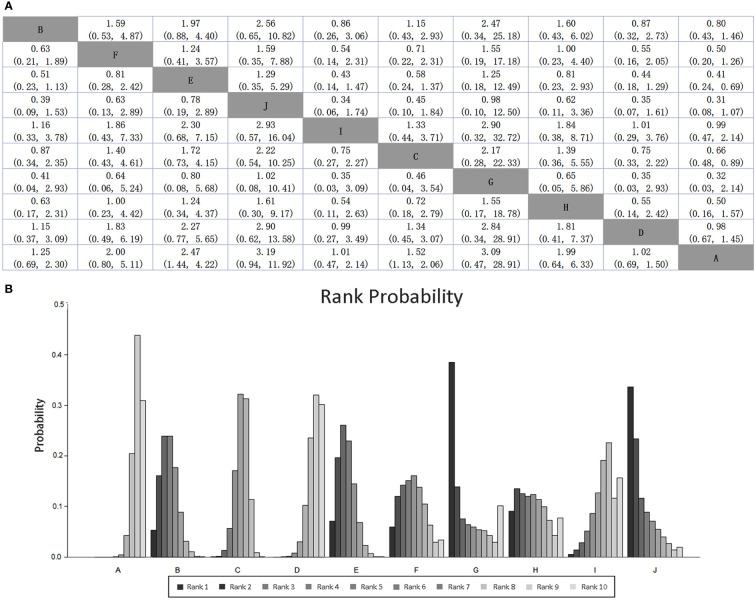
**(A)** SUCRA-based efficacy ranking league matrix showing the comparative efficacies of the regimens included in this network meta-analysis. Values below the regimens should be read from row to column, and above the treatments should be read from column to row. **(B)** Rankograms derived from relevant SUCRA values for the regimens evaluated in the included RCTs showing the cumulative rank order for each intervention. Darker color represents higher rank. Regimen labels: A: triple therapy; B: triple therapy with Bacillus; C: triple therapy with Lactobacillus; D: triple therapy with Saccharomyces; E: triple therapy with Bifidobacterium+Lactobacillus; F: triple therapy with Bacillus+Streptococcus; G: triple therapy with Lactobacillus+Propionibacterium; H: triple therapy with Lactobacillus+Streptococcus; I: triple therapy with Bifidobacterium+Lactobacillus+Streptococcus; J: triple therapy with Bifidobacterium+Lactobacillus+Saccharomyces.

Mean cure rates (95% CI) achieved by these regimens are shown in **Table 1**, and the comparative efficacy ranking league matrix, showing the comparative effect of the 10 regimens included in this NWM, is shown in **Figure 4A**. The respective rank possibility chart and the surface under the cumulative ranking curve (SUCRA) values are shown in **Figure 4B**. According to the ranking league matrix, rank possibility chart and SUCRA, the global results set manifested that triple therapy with Lactobacillus-Propionibacterium (SUCRA value 40.3%) had the best performance, with the eradication rate of 91.3% (95% CI, 78.8-103.8). Other two kinds of methods also performed well, triple therapy with Bifidobacterium-Lactobacillus-Saccharomyces reached an eradication rate of 88.2% (95% CI, 83.1-93.4; SUCRA value 34.5%), and triple therapy with Bifidobacterium-Lactobacillus has the eradication rate of 78.3% (95% CI, 75.3-81.37; SUCRA value 8.90%). Relatively, triple therapy was less effective than most of the probiotics-added therapies (eradication rate 72.8%, 95% CI, 71.4-74.2; SUCRA value 17.2%).

**Table 1:** Mean cure rates (95% CI) achieved by these regimens.

Variable	Total sample size (Responder/sample size)	Cure rates, % (95% CI)	P value
Regimen			vs TRI
TRI	2760/3792	72.8 [71.4-74.2]	–
BAC	308/365	84.4 [80.6-88.1]	P <0.05
LAC	734/920	79.8 [77.2-82.4]	P <0.05
SAC	639/877	72.9 [69.9-75.8]	P = 0.963
BIF+LAC	574/733	78.3 [75.3-81.3]	P <0.05
BAC+STR	147/176	83.5 [78.0-89.1]	P <0.05
LAC+PRO	21/23	91.3 [78.8-103.8]	P <0.05
LAC+STR	91/100	91.0 [85.3-96.7]	P <0.05
BIF+LAC+STR	290/383	75.7 [71.4-80.0]	P = 0.218
BIF+LAC+SAC	135/153	88.2 [83.1-93.4]	P <0.05

LAC, Lactobacillus; SAC, Saccharomyces; BAC, Bacillus; BIF, Bifidobacterium; STR, Streptococcus; PRO, Propionibacterium; TRI, triple therapy.

#### 3.3.3 Side effects

The direct pair comparisons (RR; 95% CI) of regimens in side effect have been shown in **Figure S8**, and all the comparisons are in **Figure S9**. Since the detail information of triple therapy with Bacillus-Streptococcus could not be found, only 9 therapies were taken into analysis. In result, Bacillus, Lactobacillus and Bifidobacterium-Lactobacillus supplement could decrease adverse effects in triple therapy significantly, whereas other kinds of therapies failed to reach significance.

**Figures S10**, **S11** show the comparative ranking league matrix and rank possibility chart, which were consistent with mean incidence of side effect in **Table 2**. Triple therapy with Lactobacillus-Propionibacterium (SUCRA value 46.6%, incidence of side effect 69.6%; 95% CI, 49.2-89.9) ranked first, representing highest side effects incidence. Triple therapy also had more chances to give rise to adverse effects, whose incidence rate was 40.3% (95% CI, 38.4-42.2). Triple therapy with Bacillus tends to have the least adverse effects among all the therapies (58.1% to rank the last, incidence rate 15.8%; 95% CI, 11.4-20.3). Bifidobacterium-Lactobacillus (30.3% probability ranking second to last in incidence rate), and Bifidobacterium-Lactobacillus-Saccharomyces supplement(incidence rate 16.9%; 95% CI, 8.3-25.4, with 25.5% probability to rank fourth in incidence rate) are also likely to have lower incidence rate, which may bring more protective effect to the eradication.

**Table 2:** Mean side effect incident rates (95% CI) achieved by these regimens.

Variable	Total sample size(Responder/sample size)	Side effect incidence, %(95% CI)	P value
Regimen			vs TRI
TRI	1070/2654	40.3 [38.4-42.2]	–
BAC	42/265	15.8 [11.4-20.3]	P <0.05
LAC	126/610	20.7 [17.4-23.9]	P <0.05
SAC	145/785	18.5 [15.8-21.2]	P <0.05
BIF+LAC	246/587	41.9 [37.9-45.9]	P = 0.477
BAC+STR	NA	NA	NA
LAC+PRO	16/23	69.6[49.2-89.9]	P <0.05
LAC+STR	61/100	61.0 [51.3-70.7]	P <0.05
BIF+LAC+STR	55/168	32.7 [25.6-39.9]	P <0.05
BIF+LAC+SAC	13/77	16.9 [8.3-25.4]	P <0.05

LAC, Lactobacillus; SAC, Saccharomyces; BAC, Bacillus; BIF, Bifidobacterium; STR, Streptococcus; PRO, Propionibacterium; TRI, triple therapy.

### 3.4 Subgroup analyses

#### 3.4.1 *H.pylori* eradication

**Table 3** shows the result of subgroup analysis for *H.pylori* eradication rate. In this study, we performed subgroup analysis to explore the effect of PPI type, antibiotic type, publication year, triple therapy duration, follow-up time, location, regimen duration and its adding time. In this way, we aimed to examine whether these factors are reflected in the outcomes of involved treatments. As displayed in **Figures S12–S19**, all the factors above did not influence on the global results, whereas analysis on antibiotic type (**Figure S15**) indicated that Tinidazole-Clarithromycin (RR = -0.01, 95% CI: -0.14‐0.12, P=0.87) and Levofloxacin-Doxycycline subgroups (RR = -0.03, 95% CI: -0.17‐0.12, P=0.70) had opposite result with the general effect (RR = 0.08, 95% CI: 0.05‐0.11, P <.01).

**Table 3:** Results of subgroup analyses for Helicobacter pylori eradication rates.

Subgroup	No. of arms	Sample size	RR (95% CI)	Peffect	I^2^(3%)	Pheterogeneity
PPI type						
Lansoprazole	10	1487	0.08 [0.03, 0.14]	<0.01	36	0.12
Esomeprazole	7	920	0.04 [-0.04, 0.13]	0.34	54	0.04
Rabeprazole	7	369	0.00 [-0.08, 0.09]	0.91	0	0.99
Omeprazole	6	1664	0.09 [0.01, 0.18]	0.03	70	<0.01
Pantoprazole	3	358	0.10 [0.02, 0.18]	0.01	0	0.47
Unclear	7	2557	0.11 [0.06, 0.16]	<0.01	59	0.02
Antibiotic type						
Amoxicillin+Clarithromycin	29	4900	0.09 [0.06, 0.12]	<0.01	45	<0.01
Tinidazole+Clarithromycin	4	159	-0.01 [-0.14, 0.12]	0.87	0	0.89
Levofloxacin+Doxycycline	2	103	-0.03 [-0.17, 0.12]	0.7	0	0.44
Amoxicillin+Moxifloxacin	1	337	0.02 [-0.08, 0.12]	0.67	NA	NA
Unclear	4	1856	0.08 [0.01, 0.16]	0.03	70	0.02
Publication year						
2000-2011	21	3488	1.10 [1.06, 1.14]	<0.01	0	0.52
2012-2022	19	3867	1.12 [1.06, 1.18]	<0.01	59	<0.01
Triple therapy duration						
7d	24	3247	1.09 [1.06, 1.13]	<0.01	0	0.78
10d	4	653	1.14 [0.95, 1.37]	0.15	72	0.01
14d	10	1927	1.10 [1.02, 1.19]	0.02	44	0.07
Unclear	2	1098	1.16 [1.04, 1.28]	<0.01	73	0.06
Follow-up time						
0-4w	9	2209	1.13 [1.07, 1.20]	<0.01	21	0.25
4-8w	26	4305	1.10 [1.05, 1.15]	<0.01	47	<0.01
>8w	2	147	1.02 [0.78, 1.33]	0.89	56	0.13
Unclear	3	694	1.11 [1.04, 1.17]	<0.01	0	0.59
Location						
Europe and America	21	2881	1.13 [1.09, 1.18]	<0.01	18	0.23
Eastern Europe	7	1395	1.10 [1.04, 1.18]	<0.01	67	<0.01
Asia	12	3079	1.10 [1.05, 1.14]	<0.01	34	0.11
Regimen duration						
<7d	12	1418	0.03 [-0.01, 0.07]	0.17	0	0.64
7-14d	12	1984	0.07 [0.00, 0.14]	0.05	69	<0.01
>14d	10	2038	0.10 [0.06, 0.14]	<0.01	0	0.85
Unclear	6	1915	0.12 [0.07, 0.17]	<0.01	34	0.18
Regimen adding time						
Before triple therapy	5	710	1.21 [1.12, 1.30]	<0.01	48	<0.01
After triple therapy	18	2609	1.08 [1.04, 1.12]	<0.01	7	0.38
With triple therapy	2	198	1.21 [1.01, 1.44]	0.04	65	0.09
Longer than triple therapy	13	2592	1.09 [1.04, 1.14]	<0.01	47	0.03
Unclear	3	1308	1.16 [1.09, 1.23]	<0.01	48	0.14

CI, confidence interval; HP, Helicobacter pylori; RR, relative risk; NA, not apply.

Stratification analysis based on probiotics duration (**Figure S12**), eradication rates can be significantly improved if the duration of antibiotic usage was longer than 14 days (RR = 0.10, 95% CI: 0.06‐0.14, P <.01), and there was significant difference between different subgroups (P < 0.05, I2 = 71.1%). Stratification analysis based on timing of probiotics addition (**Figure S13**), probiotics usage before triple therapy (RR = 1.12, 95% CI: 1.12‐1.30, P <.01) and probiotics usage after triple therapy (RR = 1.12, 95% CI: 1.01‐1.44, P <.05) can be more effective. The difference between each subgroup was statistically significant (P value of heterogeneity <0.05, I^2 = 61.4%). In the analysis of different types of antibiotics (**Figure S15**), Amoxicillin-Clarithromycin subgroup led to better consequence (RR = 0.09, 95% CI: 0.06‐0.12, P <.01). Other subgroup analysis (PPI type, publication year, triple therapy duration, follow-up time and location) didn’t show apparent difference between subgroups (**Figures S14, S16–S19**).

#### 3.4.2 Side effects

The result of subgroup analyses for side effect has been shown in **Table S4**. Among all the RCTs, 7 studies (Armuzzi et al., 2001; Ziemniak, 2006; Sung et al., 2007; Ozdil et al., 2011; Du et al., 2012; D’Angelo et al., 2014; Jin and Kim, 2019) did not contain the information of side effect.

Subgroup analysis stratified by PPI type (**Figure S20**) showed that among all kinds of PPI, rabeprazole is most effective in reducing side effects but not statistically significant (RR = -0.26, 95%CI: -0.55, 0.03, P =0.08), followed by unclear type (RR = -0.23, 95%CI: -0.28, -0.18, P <.01) and lansoprazole (RR = -0.19, 95% CI: -0.33, -0.04, P <0.05). In the analysis of effect of follow-up time (**Figure S21**), all subgroups were effective in reducing side effect, while >8w and unclear subgroup did not reach statistic significance. Significant difference could also be seen in analysis of region (**Figure S22**), as the incidence of side effect may be the lowest in Eastern Europe, whose Ratio rate was 0.38 (95% CI: 0.30, 0.49, P <.01). The incidence rate did not have special meaning in other subgroup analysis (triple therapy duration, regimen duration, regimen adding time, publication year and antibiotic type). The subgroup analysis was shown in **Figures S24–S27**.

## 4 Discussion

As a recognized carcinogen, *H.pylori* can lead to various digestive and other system diseases, and its clearance has been shown to bring better prognosis. It should be noted that after eradication, *H.pylori* positive patients can benefit from the elimination of acute gastric inflammation, reduction of chronic inflammation, improvement of peptic ulcers, thus preventing ulcer recurrence and complications, and reducing the risk of developing gastric cancer (Yang et al., 2021). Currently, the biggest hurdle of *H.pylori* treatment is antibiotic resistance (Graham and Shiotani, 2008; Graham and Dore, 2016), which cannot be solely solved by addition of antibiotics, leading to further increase in bacterial resistance to antibiotics (Graham and Dore, 2016). Therefore, we urgently need other effective methods with low side effects for *H.pylori* eradication therapy. As an emerging therapeutic enhancer, probiotics antagonize *H. pylori* by several ways, including reducing the *H.pylori* colonization density, enhancing mucosal barrier, regulating the immune response of host organisms and the secretion of anti-inflammatory cytokines and secreting compounds with broad-spectrum antimicrobial activity or metabolites(Chen et al., 2018; Goderska et al., 2018; Chen et al., 2021). It also plays an important role in reducing the fluctuation of intestinal microecology after eradication treatment, so as to improve the diversity of intestinal flora and reduce the gastrointestinal reaction caused by treatment(J. Dadashzadeh et al., 2014; Lu et al., 2016; Kafshdooz et al., 2017; Zhu and Liu, 2017; Urrutia-Baca et al., 2018; Chen et al., 2022). In previous studies, probiotics added to triple or quadruple therapy can effectively alleviate the clinical symptoms of patients with *H.pylori* infection, improve the curative effect of *H.pylori*, and reduce the incidence of adverse drug reactions. On the contrary, other studies claimed that probiotics cannot act as an aid to improve outcomes (Lü et al., 2016; McNicholl et al., 2018). These conflicting results mainly come from discrepancies in experimental design, strain types, and eradication treatment methods in different clinical trials (Dang et al., 2014; Lü et al., 2016). Since the uncertainty about its reliability, international guidelines and consensus do not strongly recommend probiotics as a routine treatment method in *H.pylori* therapy (Sugano et al., 2015; Chey et al., 2017; Liu et al., 2018). Additionally, Zhu (Zhu and Liu, 2017) found that not all probiotic or probiotics mixtures are effective during the eradication process.

This network-meta-analysis included 34 RCTs with 40 arms and 9 types of probiotic-adjuvant treatment methods identified between 2000 and 2022. The adjuvant role of different probiotics supplementation against *H.pylori* were evaluated and compared in this study. The addition of probiotics to triple therapies improved eradication rate of *H.pylori* infection (RR 1.14, 95% CI: 1.07‐1.21, P <.01), while reducing the side effects rate (RR 0.61, 95% CI: 0.53‐0.71, P <.01). The comparative efficacy of these regimens showed that Bifidobacterium-Lactobacillus and Bifidobacterium-Lactobacillus-Saccharomyces had the best comprehensive performance, which had beneficial outcome both in eradication and side effect incidence. Relatively, Lactobacillus-Propionibacterium was an effective supplement in eradication, while it also brings most side effects, which failed to reach significant comparative efficacy. Standard triple therapy was less effective than most of the probiotics added therapies when it comes to eradication rate, with the possibility of 17.2% to had the worst performance. Besides, probiotic-adjuvant regimen can increase the eradication rate and avoid adverse effects, especially given in compound preparation. As shown in the rank possibility chart, Bifidobacterium-Lactobacillus and Bifidobacterium-Lactobacillus-Saccharomyces group achieved satisfactory results both in eradication and side effect evaluation. This is possibly caused by the fact that compound probiotics can work at full capacity of different strains and is not easy to cause antibiotic resistance (Chapman et al., 2011). However, previous meta-analysis from Zhang (Zheng et al., 2013) indicating that not all combinations can bring benefits, and this may be due to the fact that the mixed strains do not show superiority in triple therapy because of the low dose of their effective strains.

In our subgroup analysis, eradication rate was closely related to follow-up time (0-4w, 4-8w), the type of antibiotics (amoxicillin+clarithromycin) and the adding time of probiotics (before or after triple therapy). Lv, et al (Lv et al., 2015) also found that the use of probiotics before or after eradication therapy could significantly increase the eradication rate compared with using probiotics at the same time. The reason may be that the use of antibiotics can easily affect the activity of living probiotics. It is recommended that the interval between probiotics and antibiotics usage had better to be more than 2 hours, whereas the existing studies have not pointed out the most suitable time for probiotics addition. Chances are that the self-protection of *H.pylori* may be activated by adding probiotics before the start of eradication therapy,while adding probiotics after the end of eradication process may cause longer medication time (Ji and Yang, 2020). The duration of probiotics is also inconclusive. >14d group had obviously better performance in our analysis. Some scholars believe that the course of probiotics should be 2 weeks (Szajewska et al., 2010; Jiang et al., 2022).

What’s more, although the short-term benefit of probiotics supplement had been revealed in the NWM, the safety of long-term application still needs to be verified by more clinical trials. At present, there are few studies on adverse reactions of probiotics (Whelan and Myers, 2010). Some studies pointed out that probiotics can induce significant adverse reactions, especially Lactobacillus and Saccharomyces, among which Saccharomyces can increase the risk of adverse events in patients with immunosuppression or life-threatening diseases, and long term use of probiotics may lead to potential risk of antibiotic resistance (Szajewska et al., 2010).

Studies have confirmed that probiotics is effective in the process of *H.pylori* treatment (Tongtawee et al., 2015; Chen et al., 2018; Plomer et al., 2020; Chen et al., 2021), while few of them evaluated the comparative effect of different kinds of probiotics. In this study, we comprehensively compared several common probiotic-adjuvant therapies and ranked their efficacy and side effects. The possible bias of RCTs in this analysis was carefully checked and excluded. What’s more, we further explored the possible influence of other factors, including region, probiotics adding time, duration of triple therapy and probiotics, follow-up time, the dosage form of antibiotics and PPI, and publication year. However, some limitations in this study should be noted. First, some therapies only included 1 or 2 studies for analysis. Some small sample studies were taken into account, and risk bias (including selection bias, performance bias, detection bias, and attrition bias) existed in a subset of studies. Second, heterogeneity cannot be neglected when combining and analyzing data from different studies. For example, the dosage of antibiotics and probiotics, the dosage form of probiotics, antibiotics resistance status of *H.pylori*, and patient compliance might lead to the existence of heterogeneity. However, we were not able to divide these data into different subgroups for further analysis due to the lack of relevant information from the primary studies, or not enough sample number for each subgroup. Besides, some probiotics regimen in this analysis were given in a mixture, containing other component like yogurt or antioxidant, which may also have impact on the results of eradication and side effects. Fourth, not all the studies included the information of side effect rates, and the severity of the adverse events was not evaluated in the present study. Last but not least, this study conducted a network comparison including both direct and indirect pairs, and is free of direct clinical data to support the promising efficacy of probiotics supplement in anti-*H.pylori* treatment, so its validity and clinical value should be further explored by future clinical trials.

## 5 Conclusion

Compared to traditional triple therapy, the addition of probiotics can improve eradication rates and reduce side effects. The comparative effectiveness ranking results showed that Bifidobacterium-Lactobacillus therapy and Bifidobacterium-Lactobacillus-Saccharomyces therapy had relatively better performance when considering the comprehensive outcome in eradication rate and side effect incidence. Probiotics adding before or after triple therapy, and duration of probiotics longer than 14 days can also improve therapeutic effect, but the reliability of this view needs to be further confirmed. Combined usage of different probiotics, although more effective compared to single usage of probiotics, was tested in few studies and more research from various parts of the world are needed.

## Author contributions

YW and LM designed the meta-analysis. YW and XW performed the literature search and drafted the manuscript. X-YC and H-LZ screened the literature and assessed the quality. X-YC, YW, and XW extracted data. YW and XW analyzed and interpreted the data. and LM and H-LZ proofread the paper. All authors contributed to the article and approved the submitted version.
