# Supplementary material for: Comparative effectiveness of different probiotics supplements for triple helicobacter pylori eradication: a network meta-analysis
Source: Front Cell Infect Microbiol. 2023 May 15;13:1120789. doi: 10.3389/fcimb.2023.1120789 (PMC10226649; doi:10.3389/fcimb.2023.1120789)
Supplement: Supplementary file 1 [file Table_1.doc]

Supplementary Material

# Comparative Effectiveness of Different Probiotics Supplements for Triple Helicobacter pylori eradication: A Network Meta-analysis

**Yue Wang^†1^, Xue Wang^†1^, Xue-Yan Cao^1^, Han-Long Zhu^2^, Lin Miao^1^**

^1^Medical Centre for Digestive Diseases, the Second Affiliated Hospital of Nanjing Medical University, Nanjing 210011, Jiangsu Province, China

^2^Department of Gastroenterology and Hepatology, Jinling Hospital, Affiliated Hospital of Medical School, Nanjing University, Nanjing, Jiangsu, China.

*** Correspondence:** Lin Miao: linmiao@njmu.edu.cn.

# **1** Supplementary Figures and Tables

## Supplementary Figures


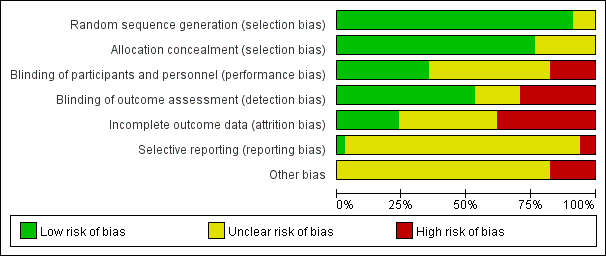


**Supplementary Figure 1.** Risk of bias graph: review authors' judgement about each risk of bias item presented as percentages across all included studies.


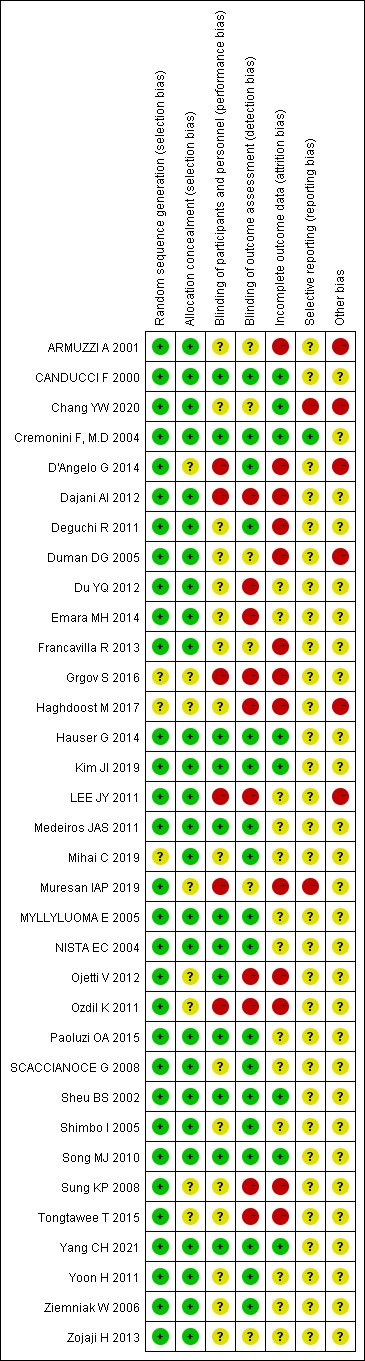


**Supplementary Figure 2.** Risk of bias summary: review authors' judgement about each risk of bias item for each included research. “+” indicates a low risk of bias, , “-”indicates a high risk of bias, “?” indicates unclear a risk of bias.


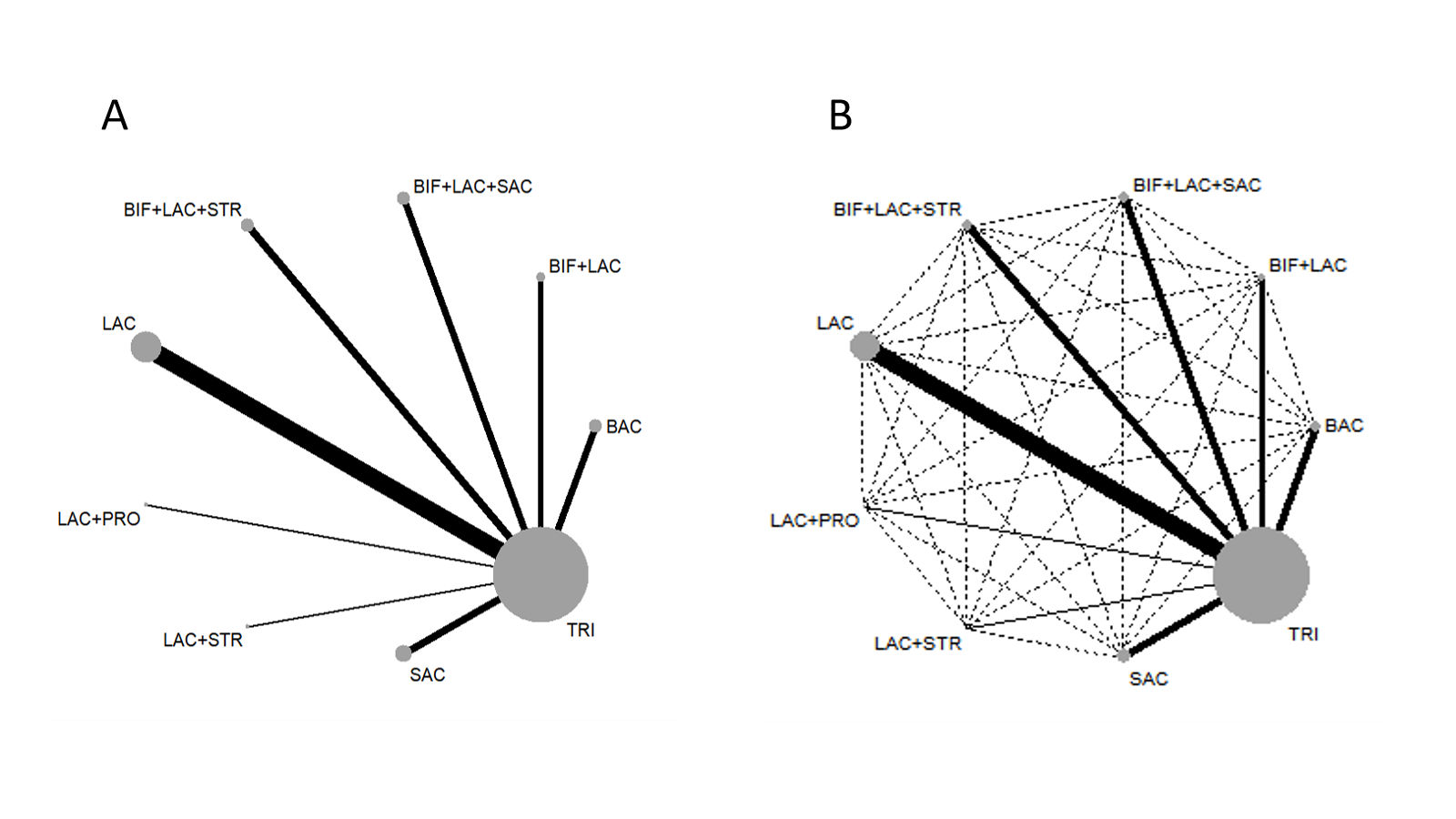


**Supplementary Figure 3.** Network map of direct comparisons of side effect included in all the RCTs.

(A) Network map of the 8 direct comparisons included in all the RCTs. The node size reflects the number of patients allocated to each treatment, whereas edge thickness is in proportion to the precision, which is the inverse of variance of each direct comparison. (B) Network map of all 36 comparisons in this NWM, including 8 direct (solid lines) and 28 indirect (interrupted lines).

Abbreviations: LAC, Lactobacillus; SAC, Saccharomyces; BAC, Bacillus; BIF, Bifidobacterium; STR, Streptococcus; PRO, Propionibacterium; TRI, triple therapy.


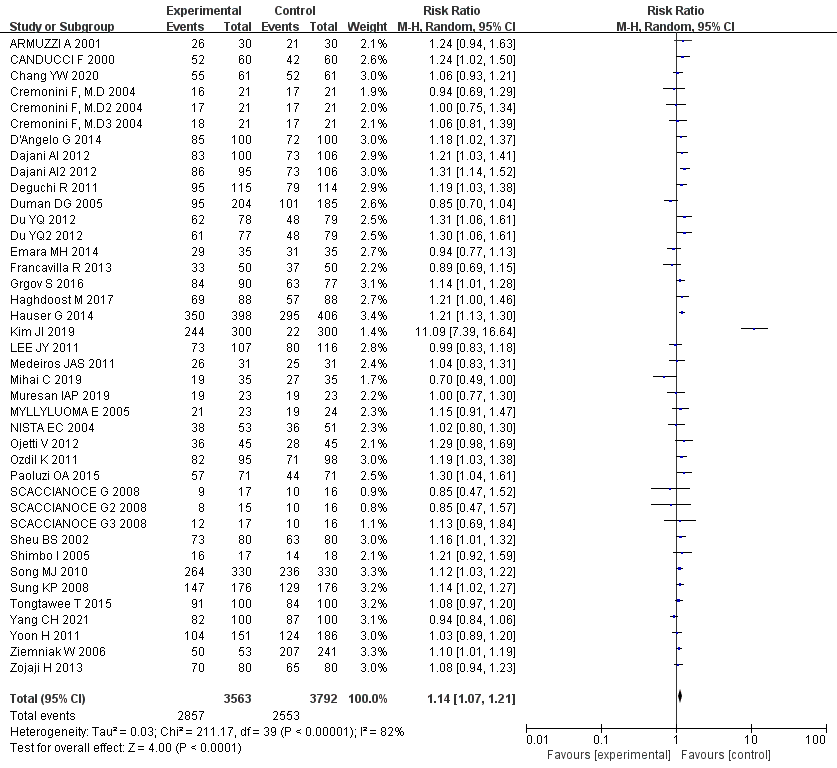


**Supplementary Figure 4.** Forest plot for effects of overall comparison result in eradication.


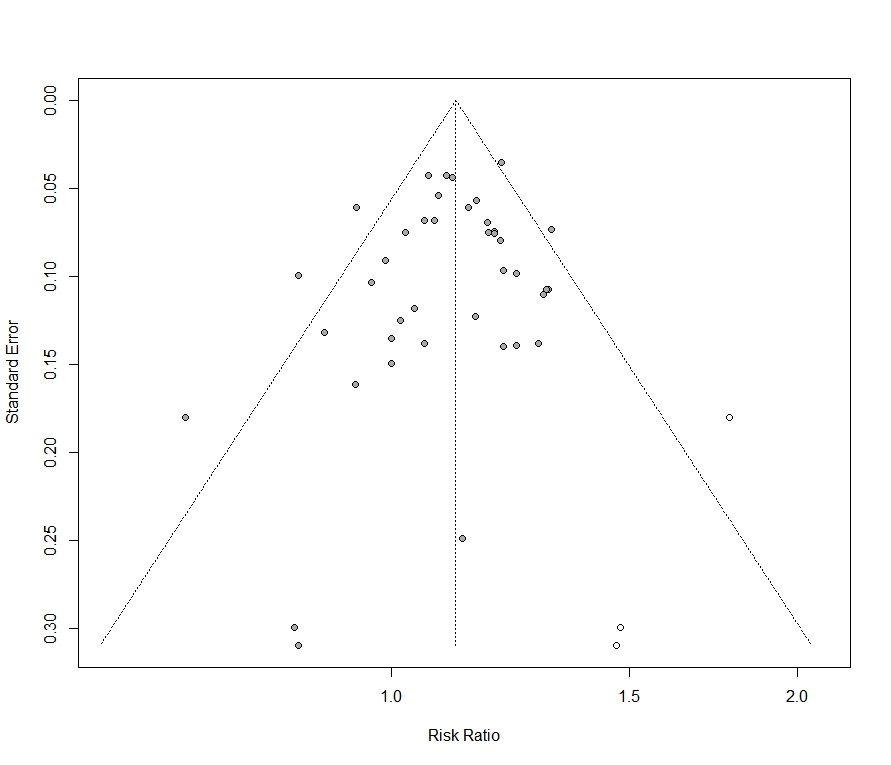


**Supplementary Figure 5.** Begg's funnel plot for overall *H.pylori* eradication rates.


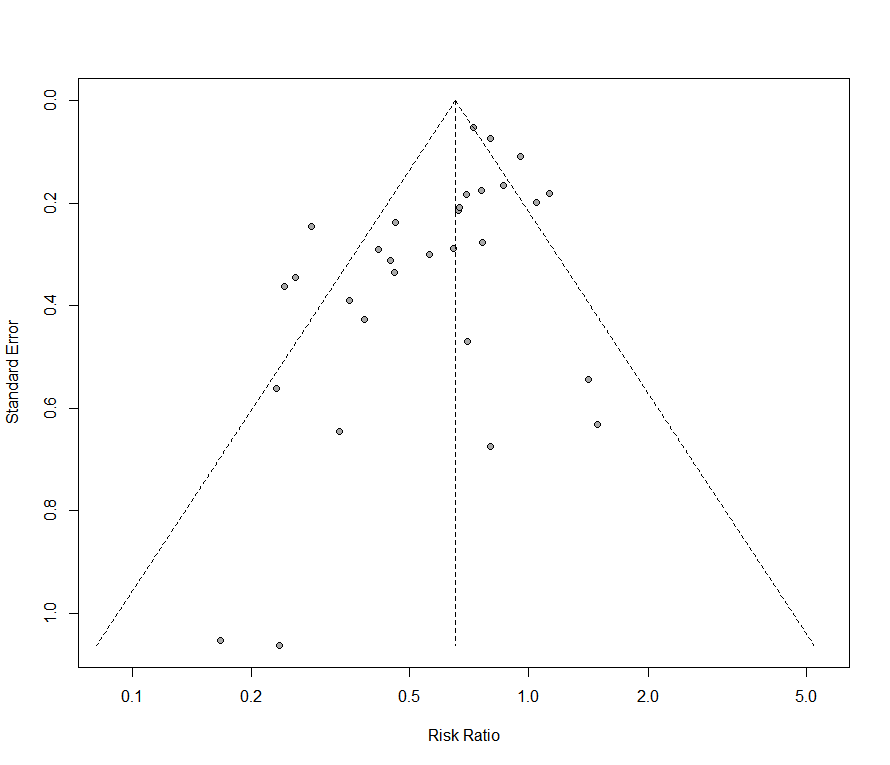


**Supplementary Figure 6.** Begg's funnel plot for overall *H. pylori* side effect.


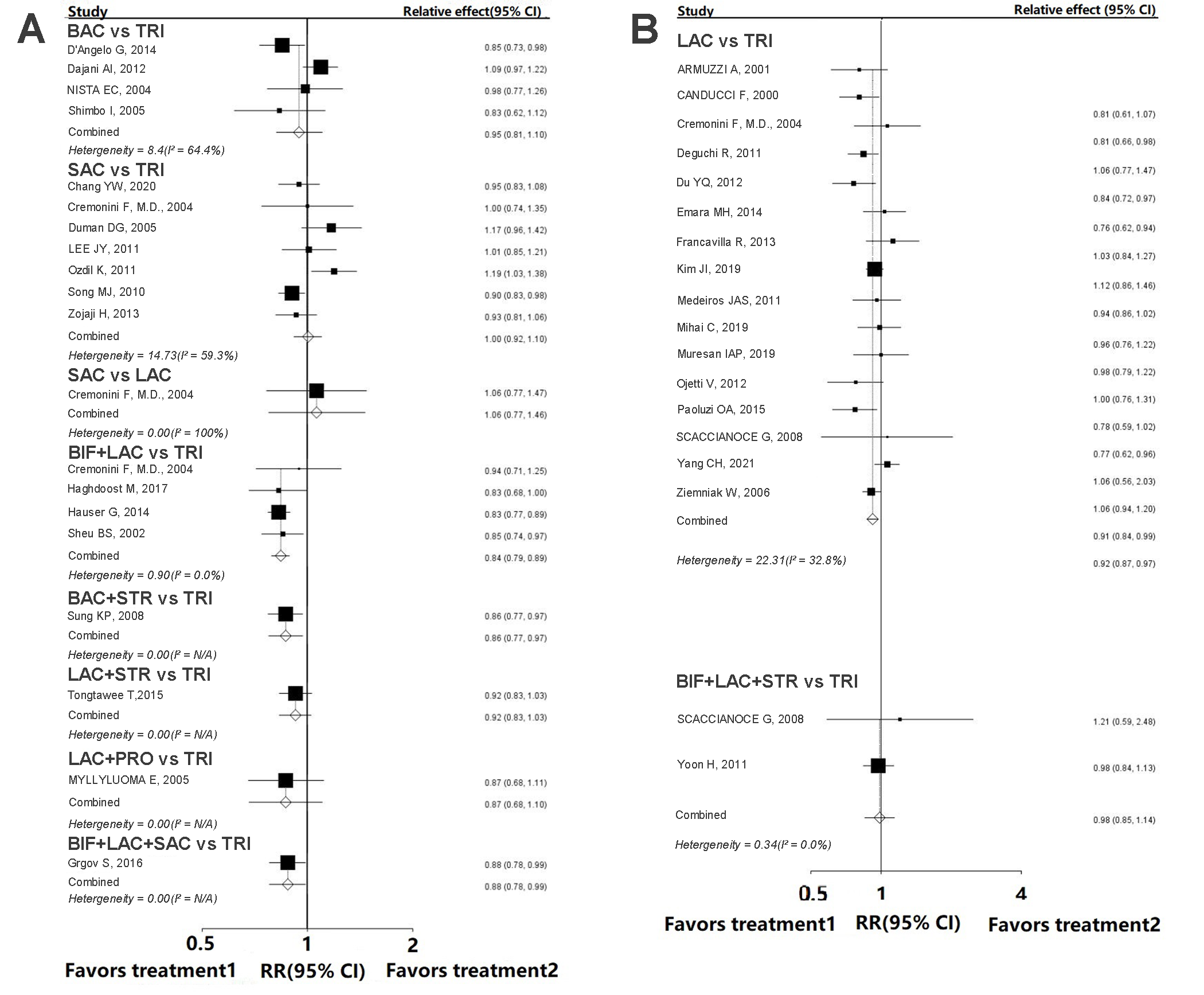


**Supplementary Figure 7.** Forest plot for direct comparisons of eradication rate. Network forest plot illustrating all included pair comparisons grouped in 10 comparisons (RR; 95% CI) of regimens included in the RCTs in eradication rate.

Abbreviations: BAC, Bacillus; BIF, Bifidobacterium; LAC, Lactobacillus; PRO, Propionibacterium; SAC, Saccharomyces; STR, Streptococcus; TRI, triple therapy.


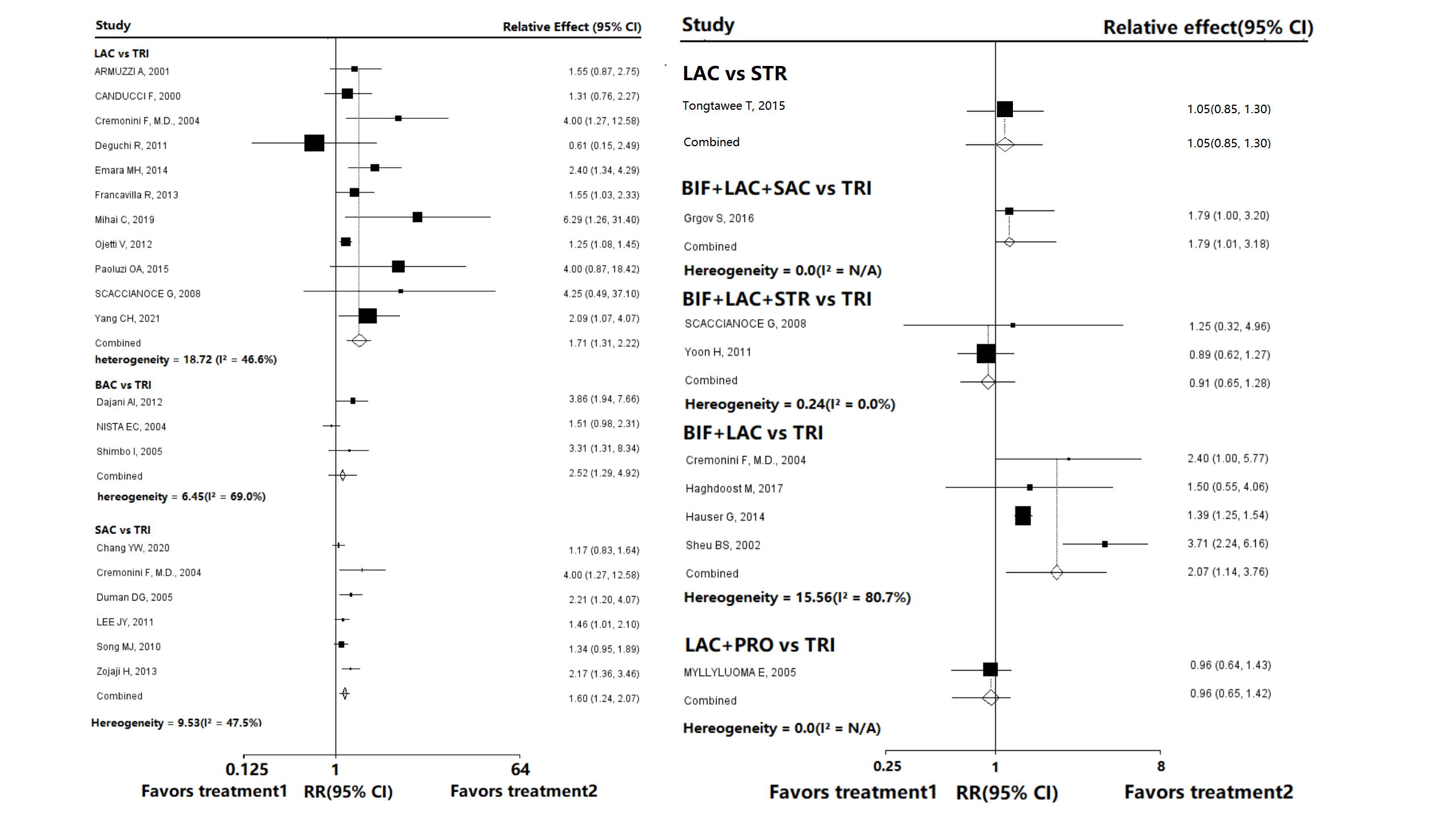


**Supplementary Figure 8.** Forest plot for direct comparisons of adverse effects.

Abbreviations: BAC, Bacillus; BIF, Bifidobacterium; LAC, Lactobacillus; PRO, Propionibacterium; SAC, Saccharomyces; STR, Streptococcus; TRI, triple therapy.


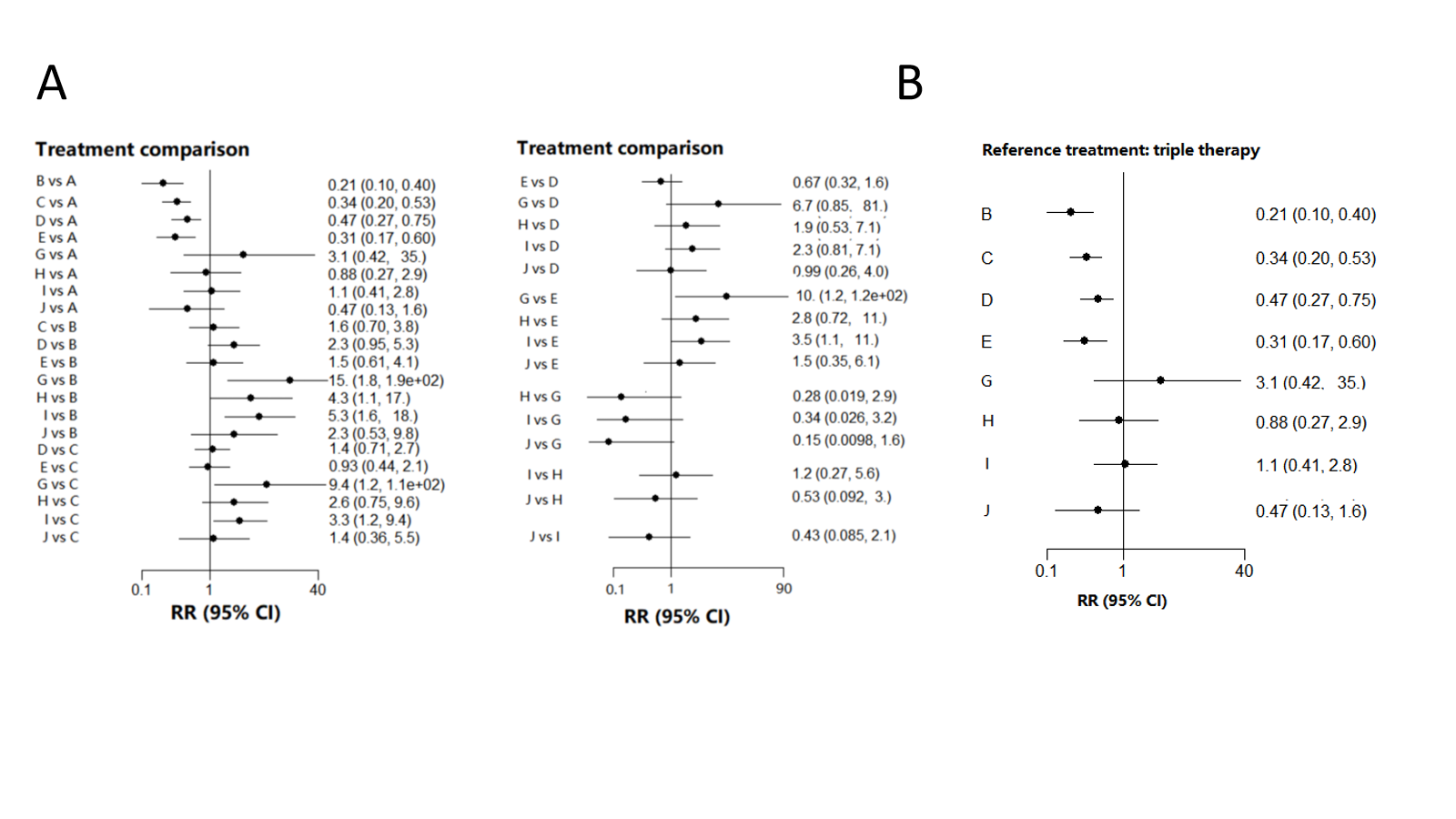


**Supplementary Figure 9.** Forest plot for all comparisons of adverse effects. (A)Forest plot (RR; 95% CI) illustrating all direct/indirect 36 side effect pair comparisons of regimens included in all the RCTs. (B) Forest plot showing the incidence of adverse effect of the regimens compared directly with the triple therapy reference regimen.

Abbreviations: RR, risk ratio; Regimen labels: A: triple therapy; B: triple therapy with Bacillus; C: triple therapy with Lactobacillus; D: triple therapy with Saccharomyces; E: triple therapy with Bifidobacterium+Lactobacillus; F: triple therapy with Bacillus+Streptococcus; G: triple therapy with Lactobacillus+Propionibacterium; H: triple therapy with Lactobacillus+Streptococcus; I: triple therapy with Bifidobacterium+Lactobacillus+Streptococcus; J: triple therapy with Bifidobacterium+Lactobacillus+Saccharomyces.


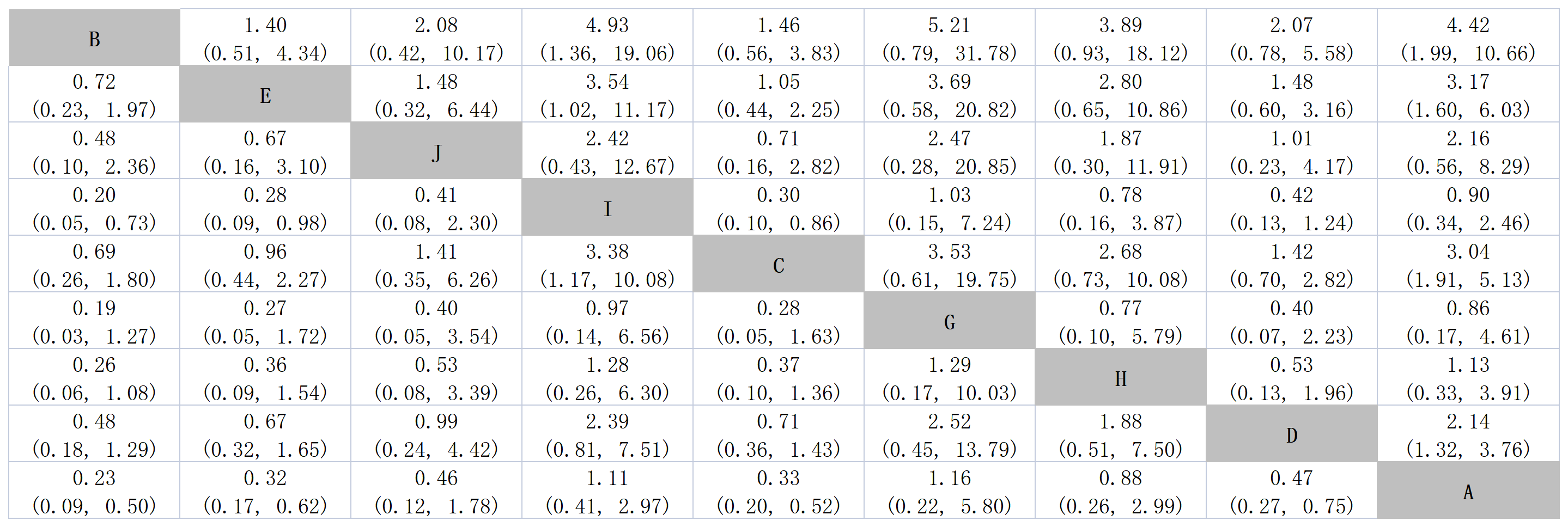


**Supplementary Figure 10.** SUCRA-based efficacy ranking league matrix showing the comparative incidence of adverse effect of the regimens included in this network meta-analysis.

Abbreviations: Regimen labels: A: triple therapy; B: triple therapy with Bacillus; C: triple therapy with Lactobacillus; D: triple therapy with Saccharomyces; E: triple therapy with Bifidobacterium+Lactobacillus; F: triple therapy with Bacillus+Streptococcus; G: triple therapy with Lactobacillus+Propionibacterium; H: triple therapy with Lactobacillus+Streptococcus; I: triple therapy with Bifidobacterium+Lactobacillus+Streptococcus; J: triple therapy with Bifidobacterium+Lactobacillus+Saccharomyces.


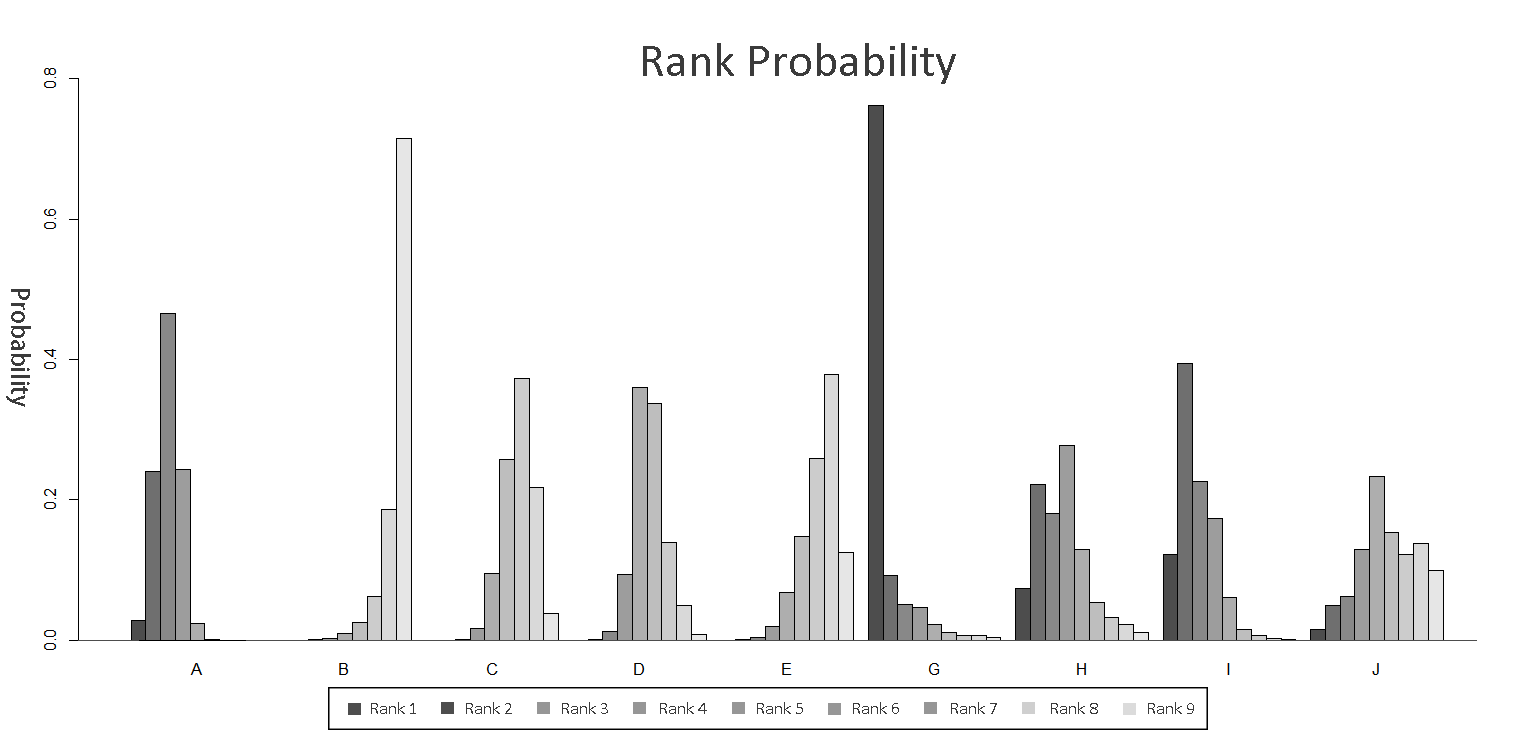


**Supplementary Figure 11.** Rankograms derived from relevant SUCRA values for the regimens evaluated in the included RCTs showing the cumulative rank order of incidence of side effect(1–9).

Regimen labels: A: triple therapy; B: triple therapy with Bacillus; C: triple therapy with Lactobacillus; D: triple therapy with Saccharomyces; E: triple therapy with Bifidobacterium+Lactobacillus; G: triple therapy with Lactobacillus+Propionibacterium; H: triple therapy with Lactobacillus+Streptococcus; I: triple therapy with Bifidobacterium+Lactobacillus+Streptococcus; J: triple therapy with Bifidobacterium+Lactobacillus+Saccharomyces.


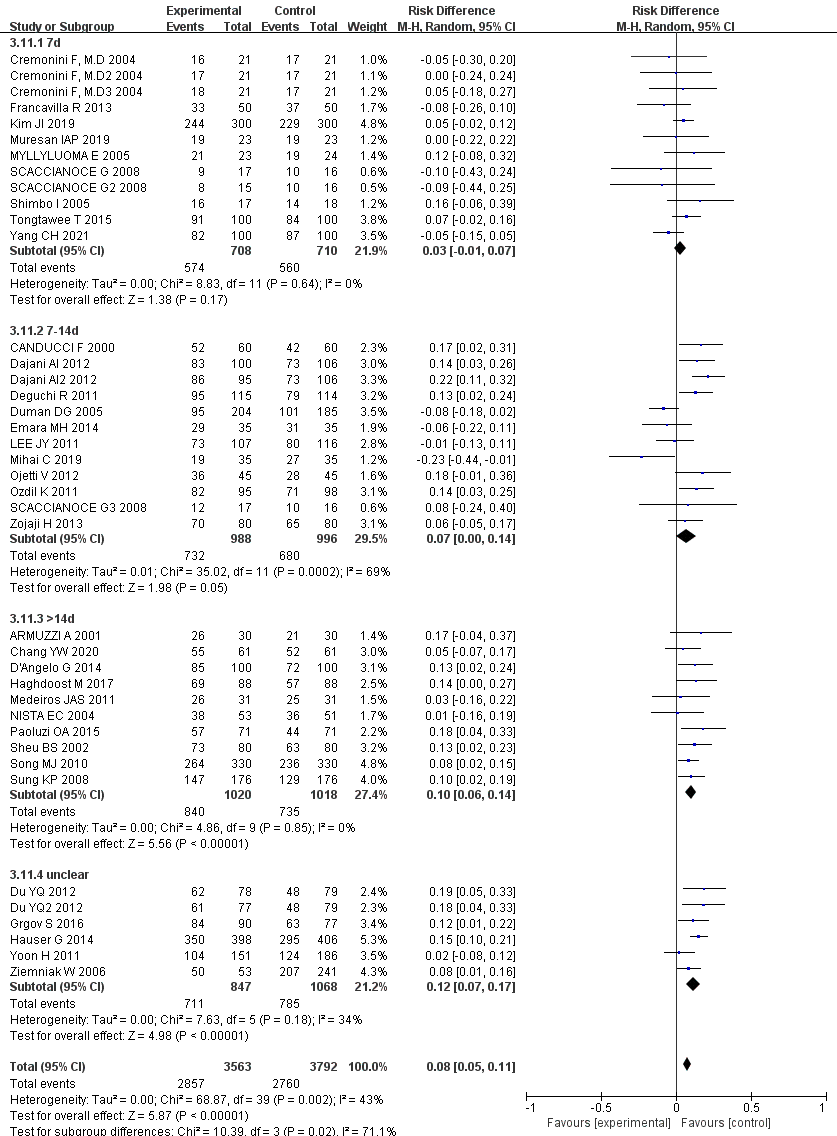


**Supplementary Figure 12.** Subgroup analysis for *H.pylori* eradication rates based on probiotics duration.


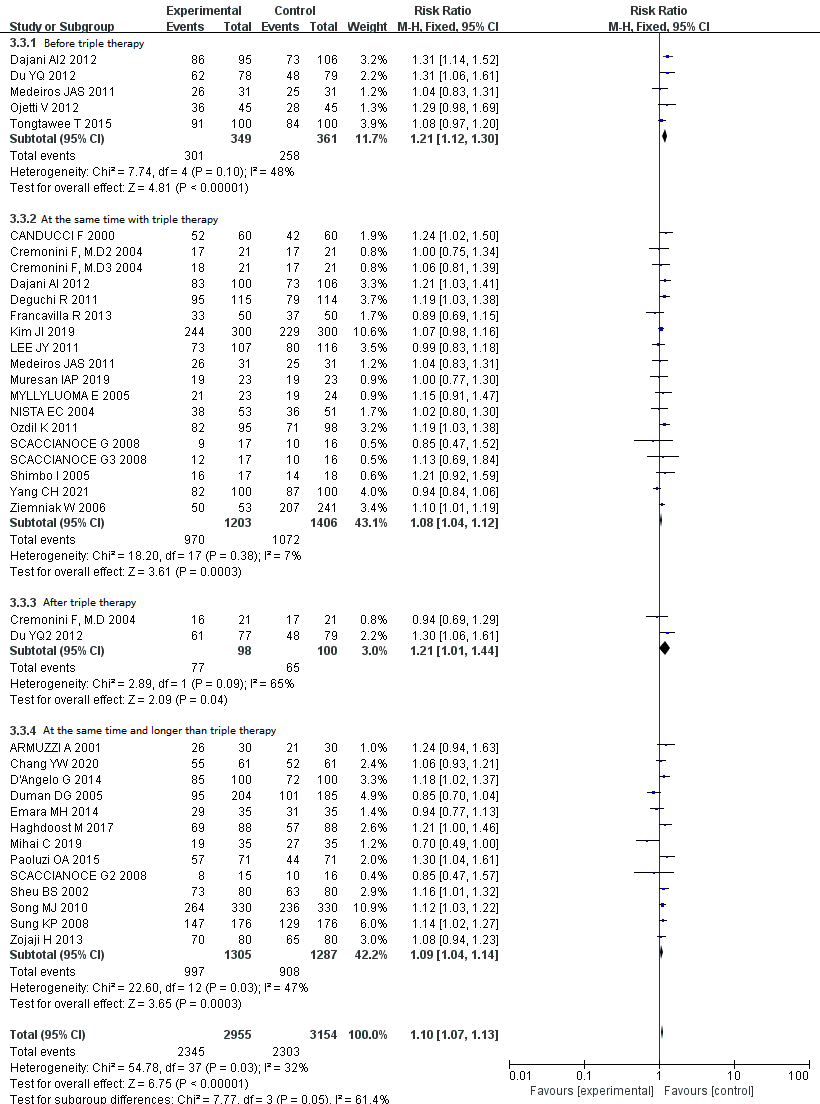


**Supplementary Figure 13.** Subgroup analysis for *H.pylori* eradication rates based on timing of probiotics addition.


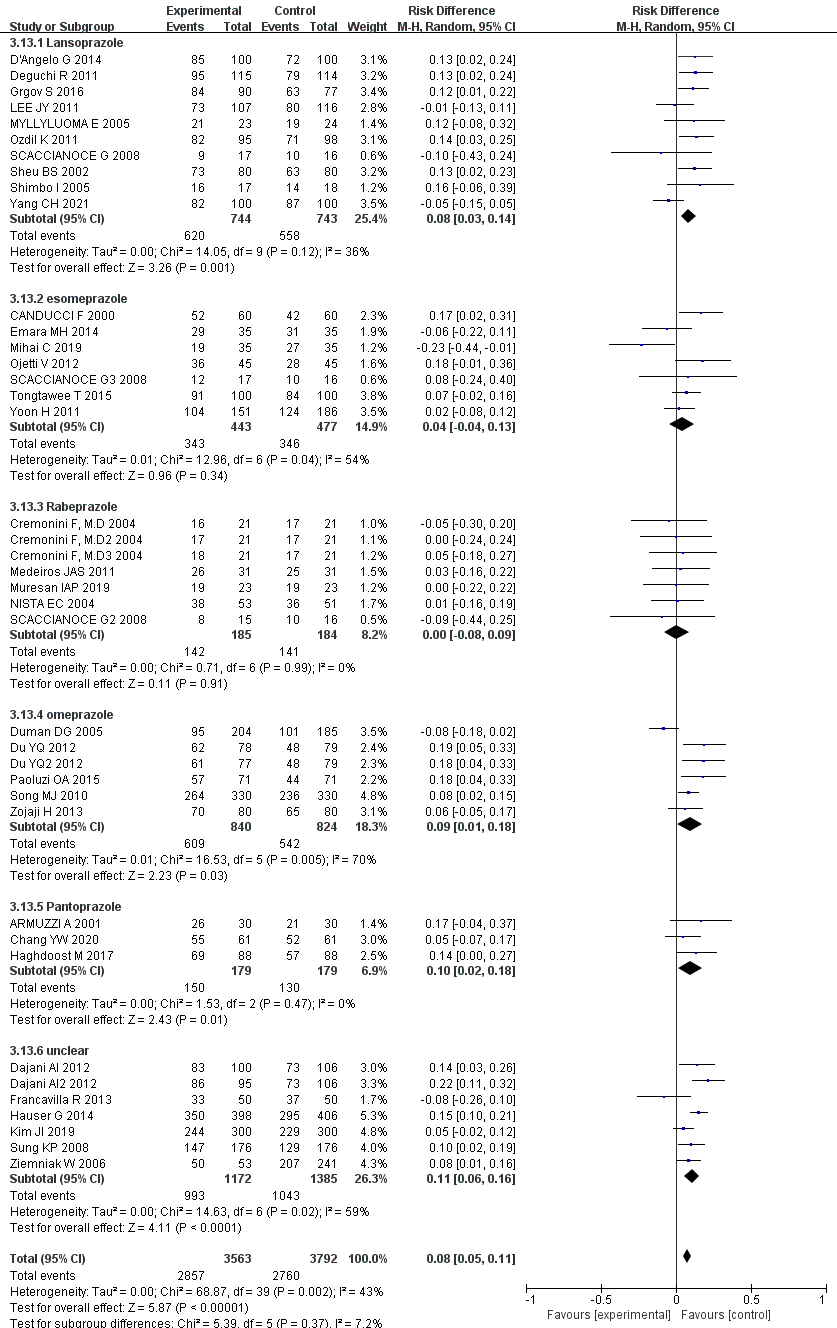


**Supplementary Figure 14.** Subgroup analysis for *H.pylori* eradication rates based on PPI type.


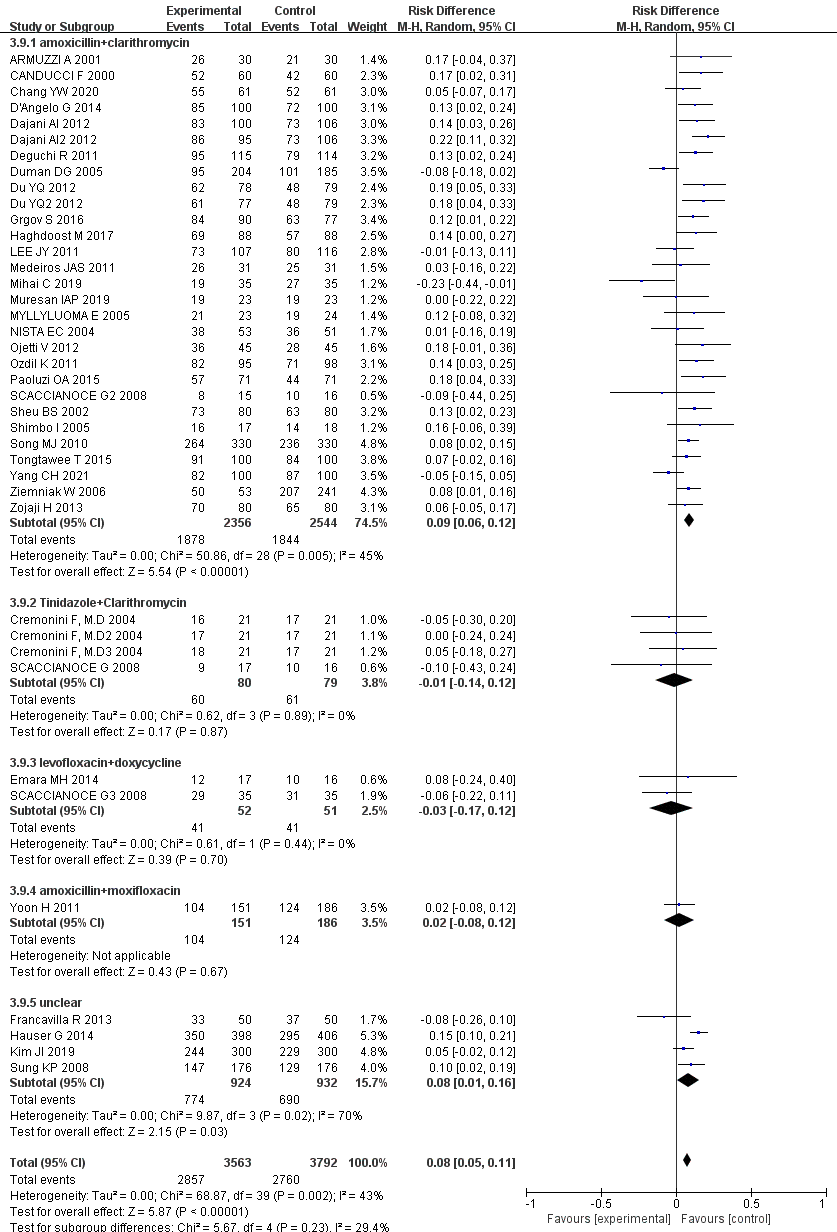


**Supplementary Figure 15.** Subgroup analysis for *H.pylori* eradication rates based on antibiotic type.


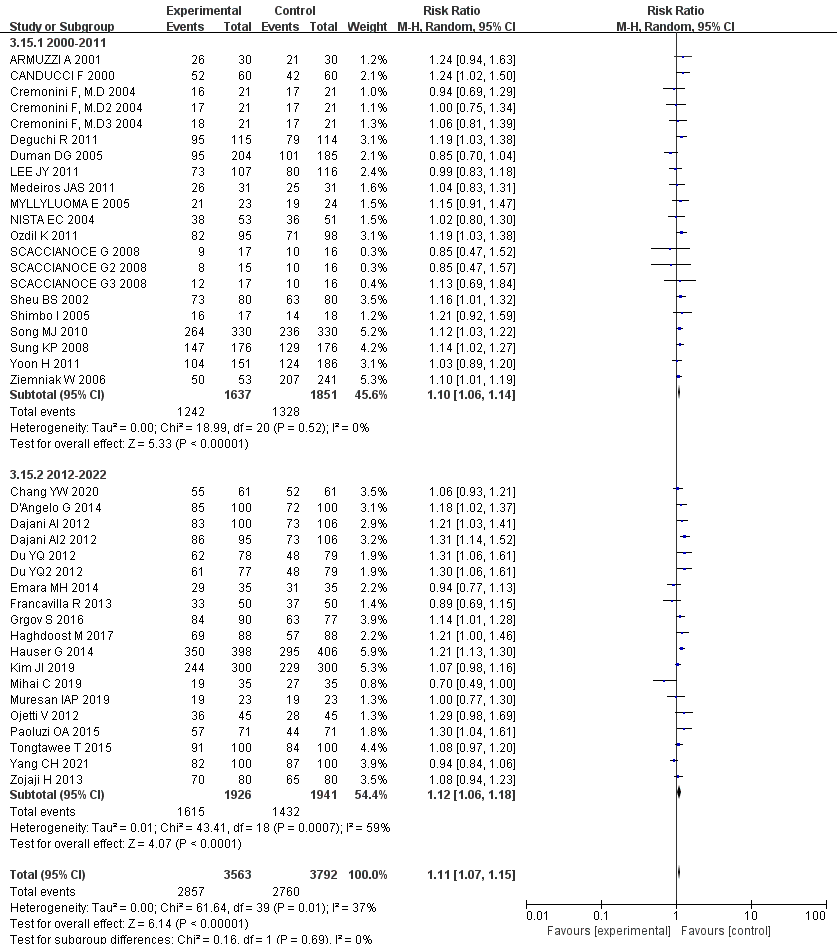


**Supplementary Figure 16.** Subgroup analysis for *H.pylori* eradication rates based on publication year.


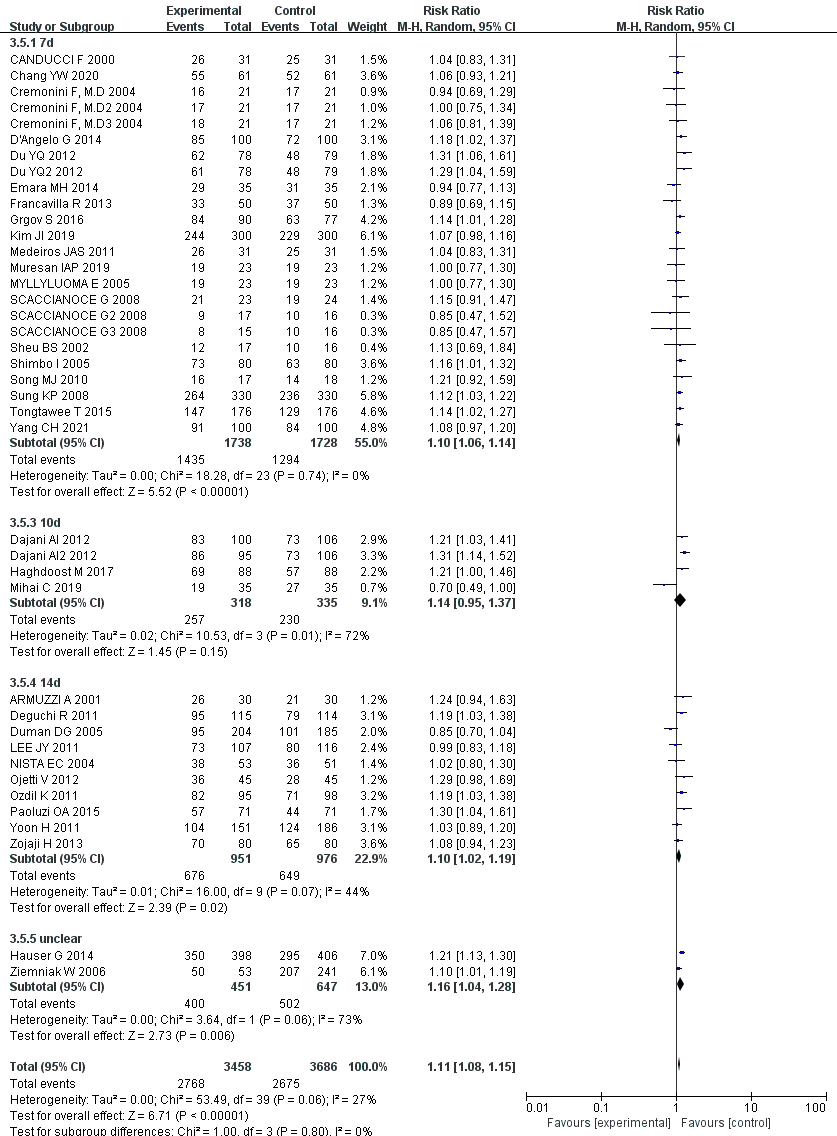


**Supplementary Figure 17.** Subgroup analysis for *H.pylori* eradication rates based on triple therapy duration.


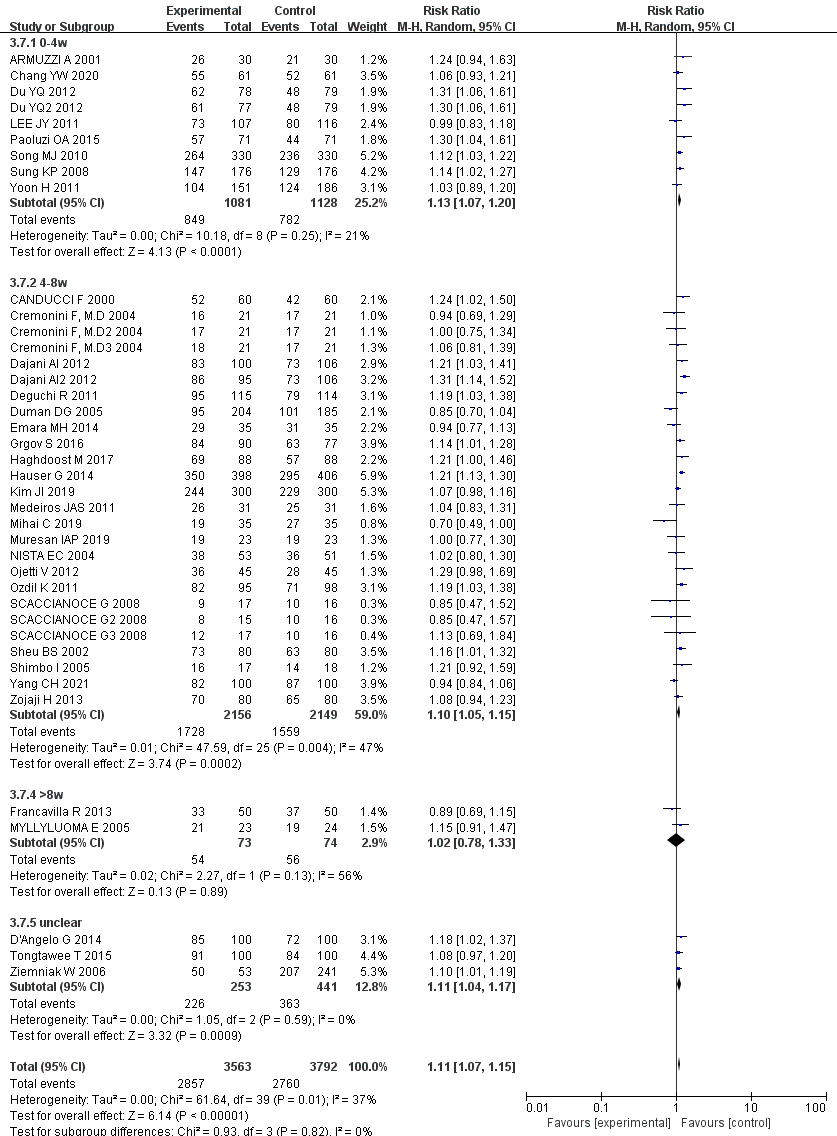


**Supplementary Figure 18.** Subgroup analysis for *H.pylori* eradication rates based on follow-up time.


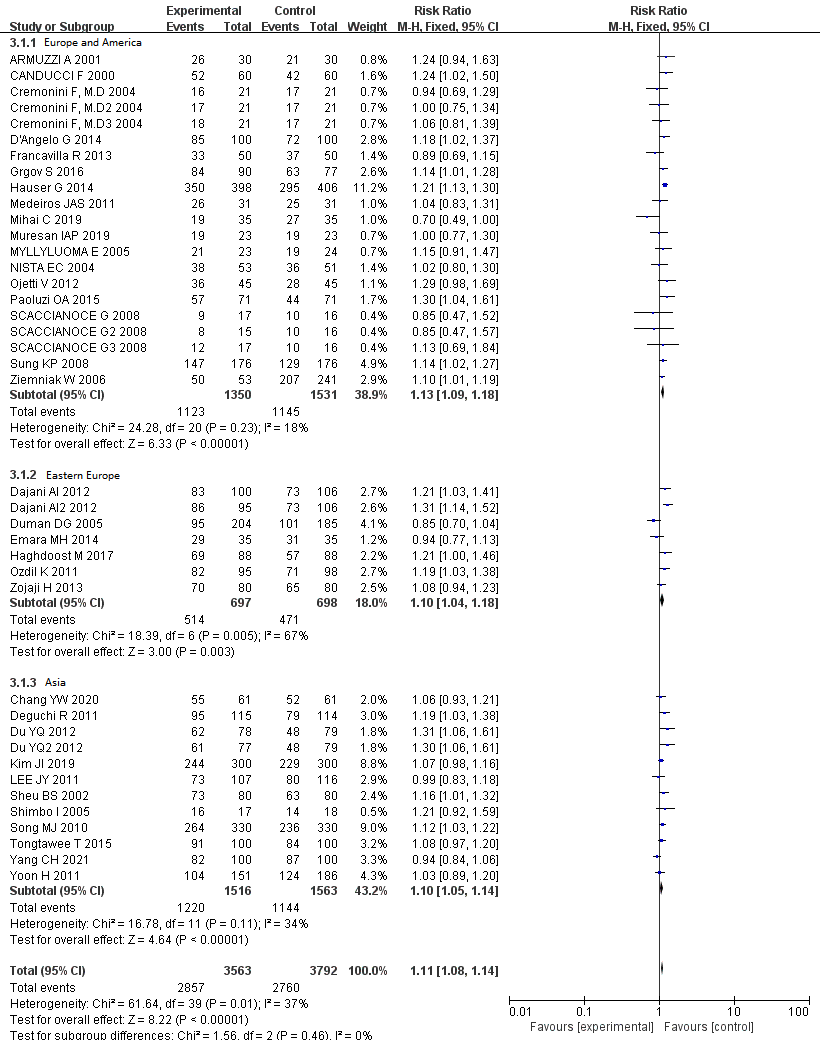


**Supplementary Figure 19.** Subgroup analysis for *H.pylori* eradication rates based on publication location.


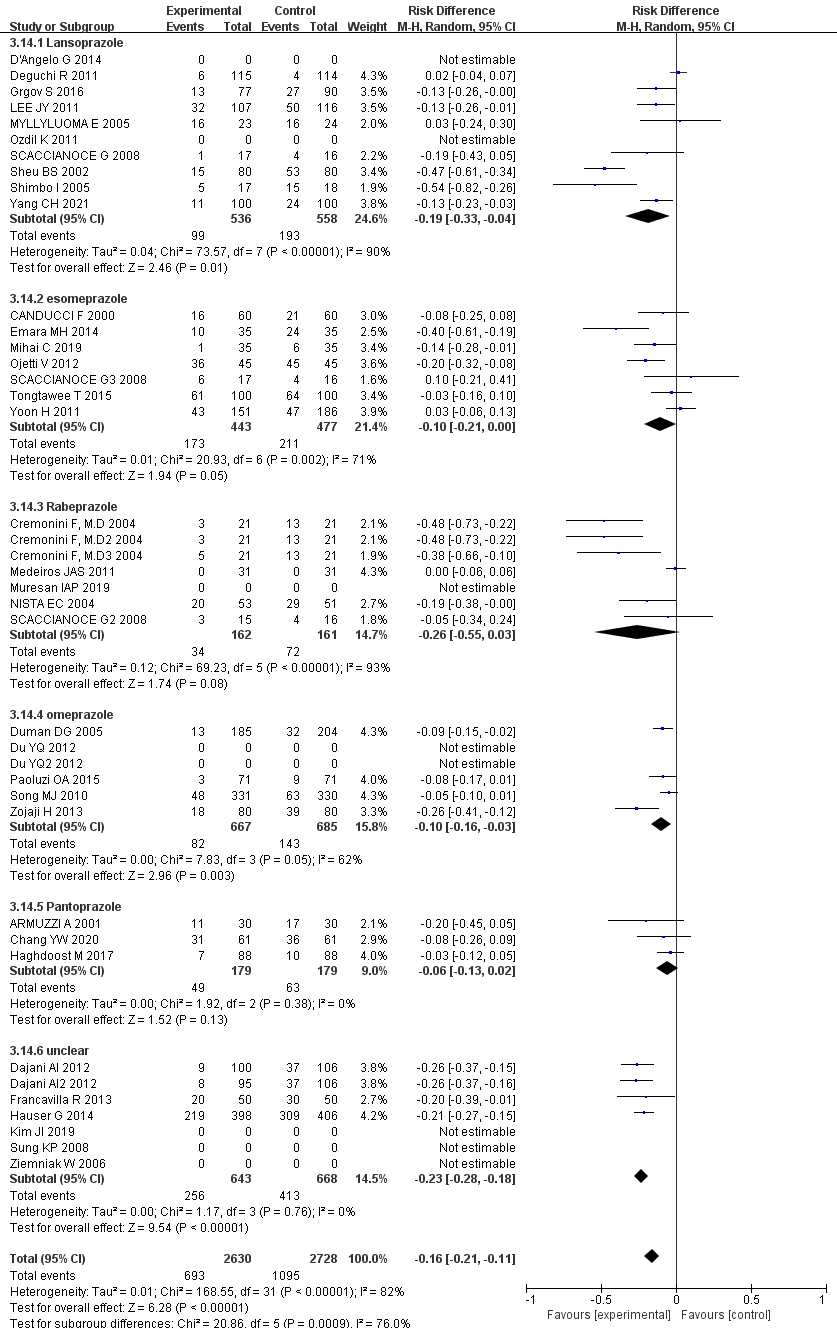


**Supplementary Figure 20.** Subgroup analysis for *H.pylori* side effect based on PPI type.


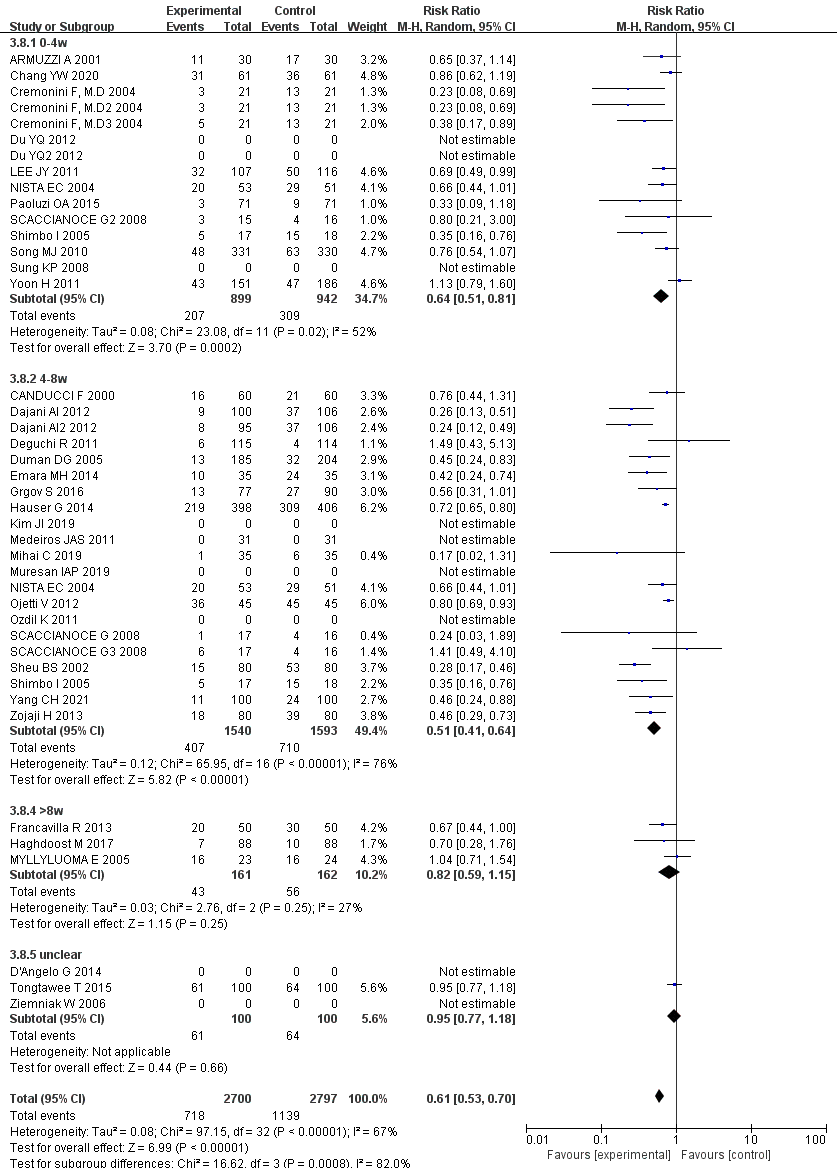


**Supplementary Figure 21.** Subgroup analysis for *H.pylori* side effect based on follow-up time.


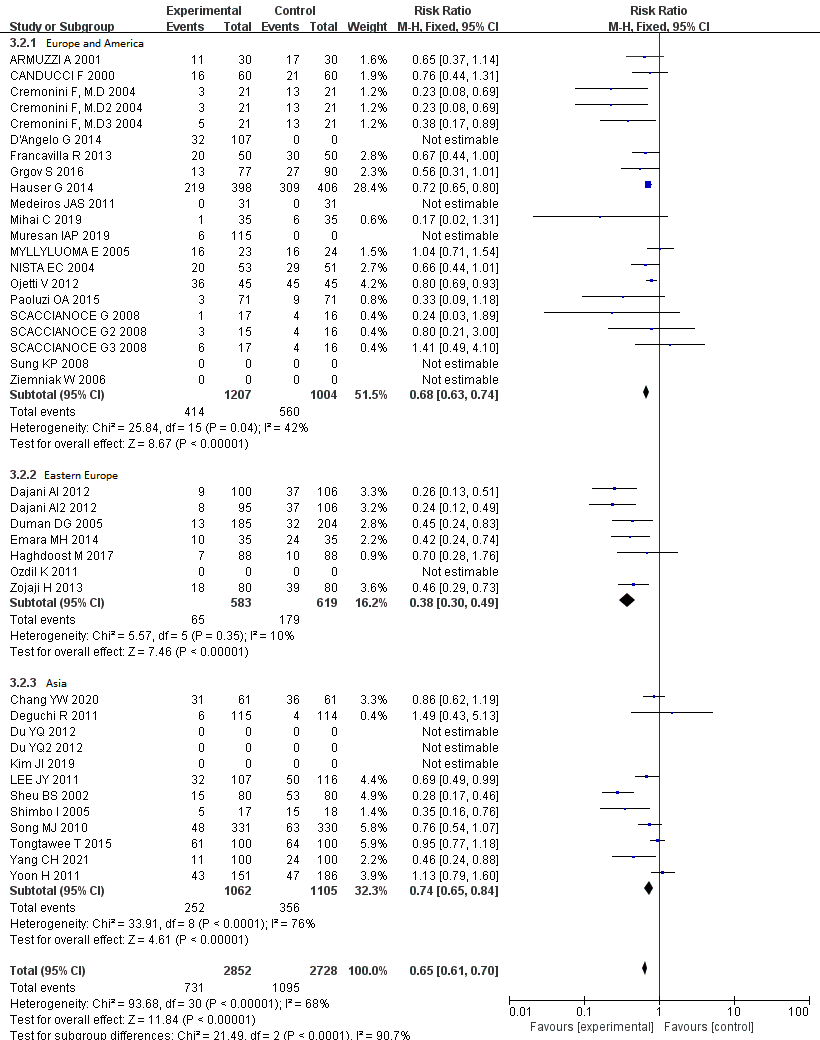


**Supplementary Figure 22.** Subgroup analysis for *H.pylori* side effect based on location.


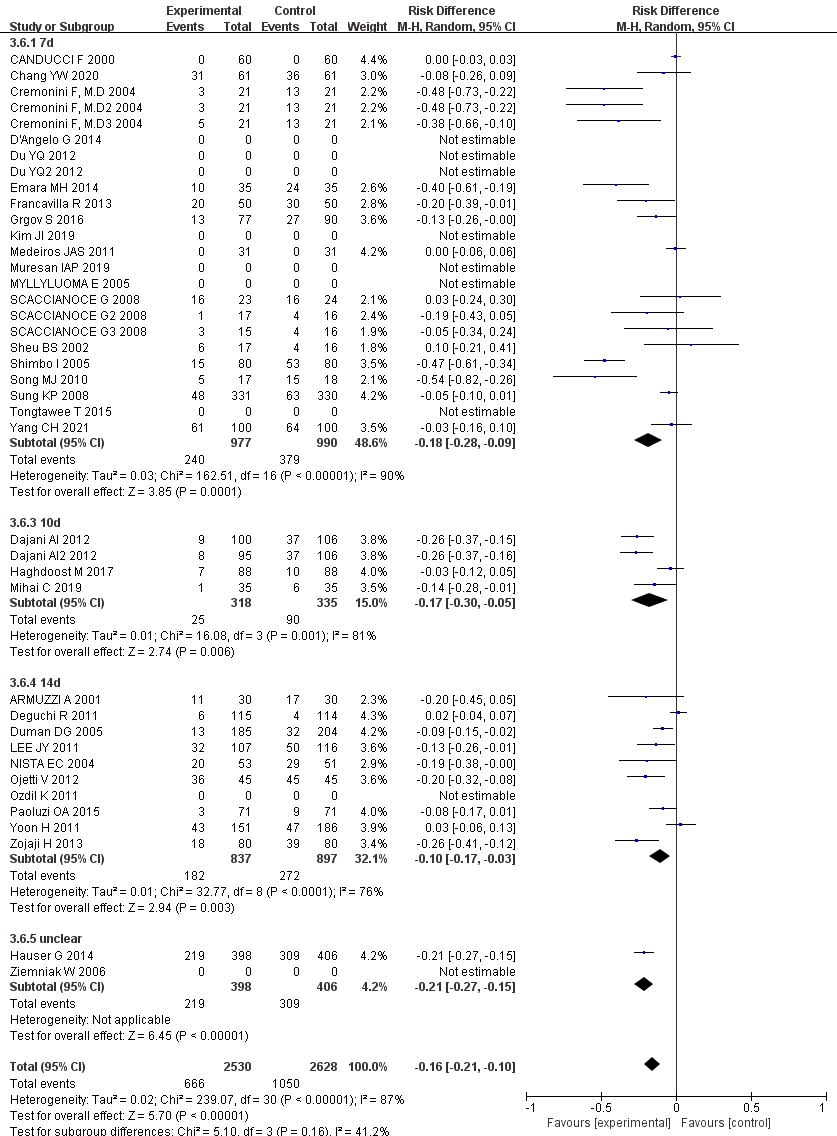


**Supplementary Figure 23.** Subgroup analysis for *H.pylori* side effect based on triple therapy duration.


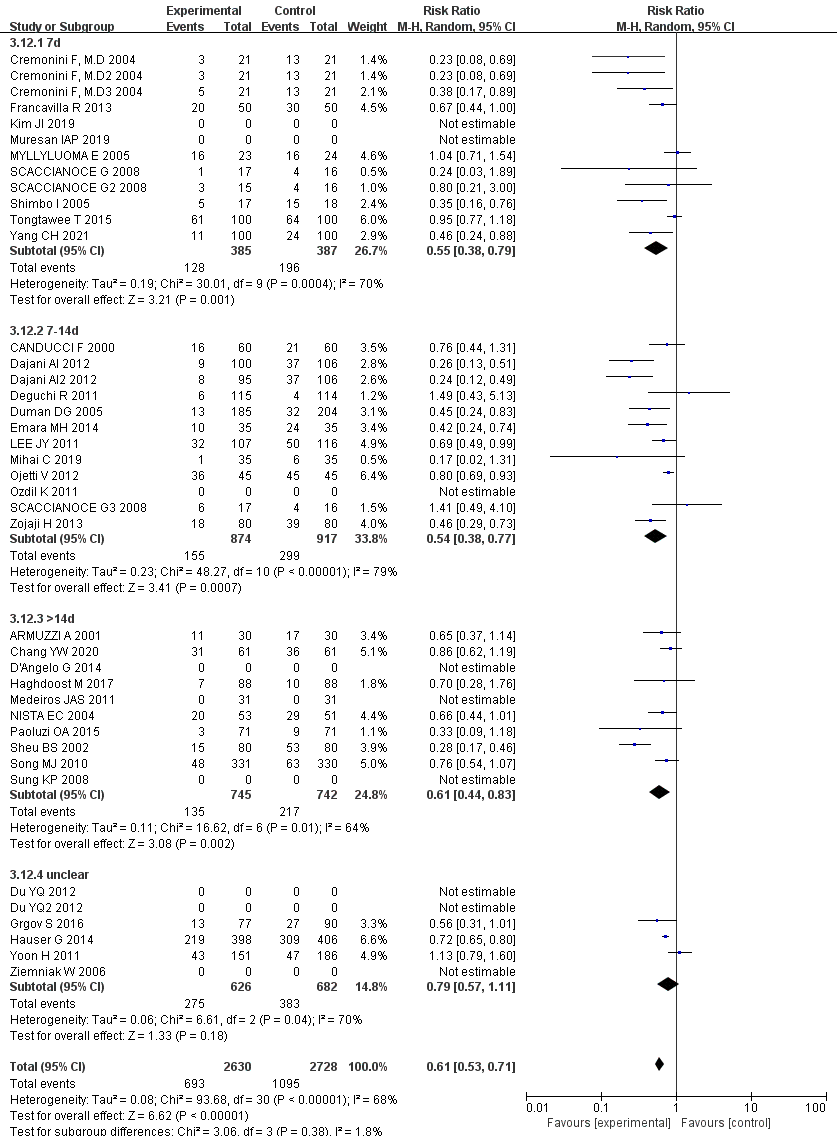


**Supplementary Figure 24.** Subgroup analysis for *H.pylori* side effect based on probiotics duration.


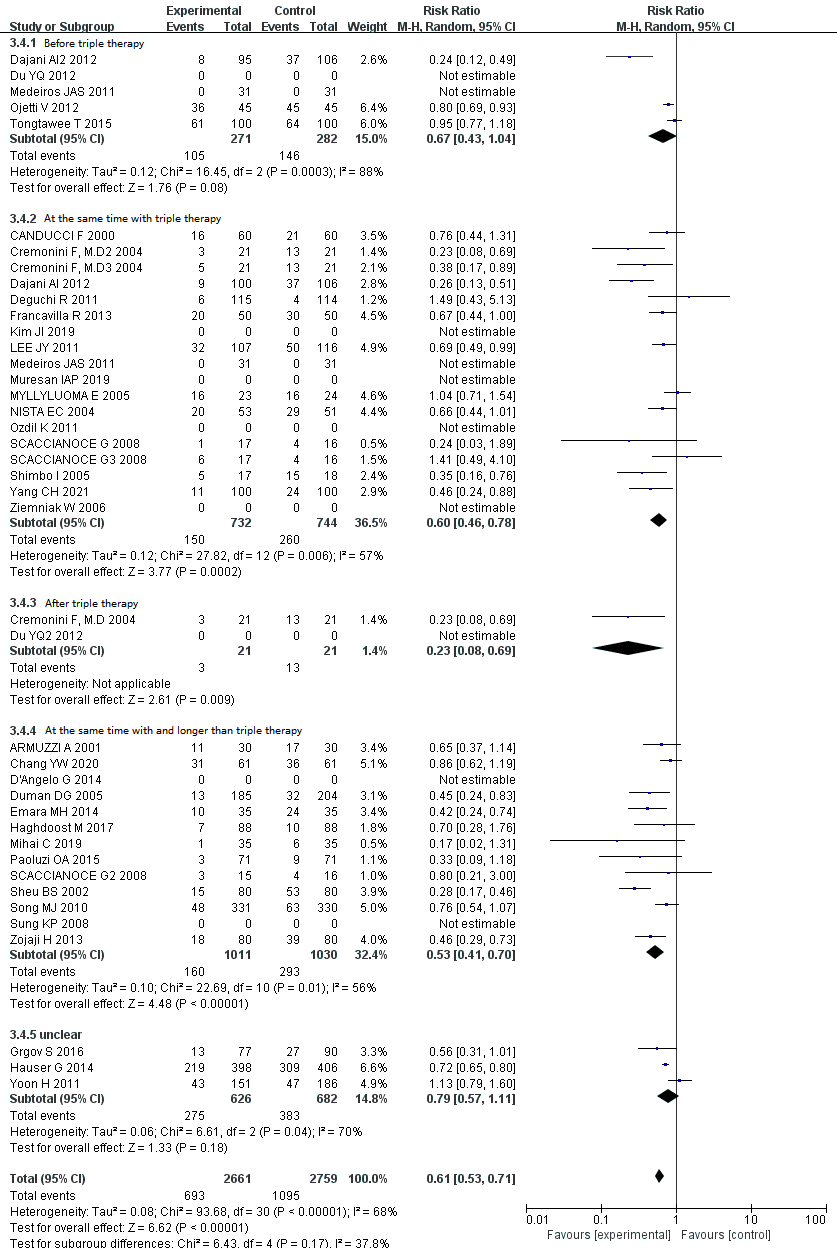


**Supplementary Figure 25.** Subgroup analysis for *H.pylori* side effect based on probiotics adding time.


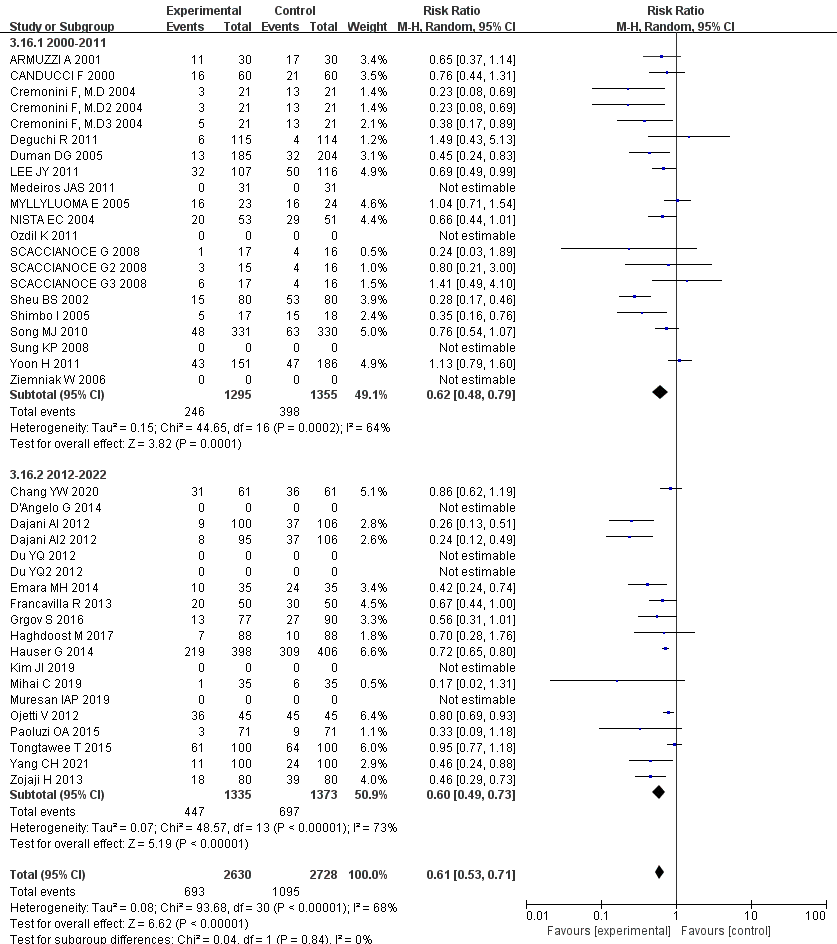


**Supplementary Figure 26.** Subgroup analysis for *H.pylori* side effect based on publication year.


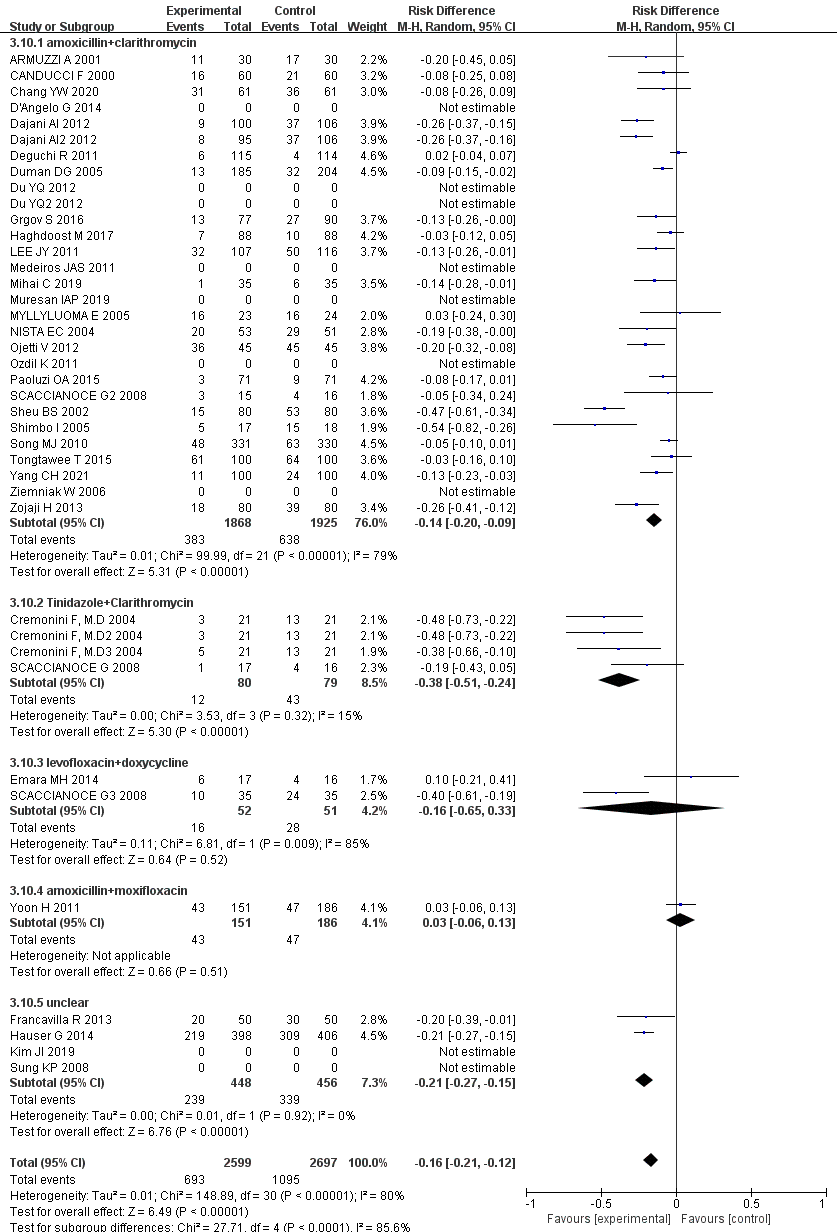


**Supplementary Figure 27.** Subgroup analysis for *H.pylori* side effect based on antibiotic type.

## Supplementary Tables

Table S1 Search Strategy (from inception to April 2022)

| **Database** | **Search strings** | **Results** |
| --- | --- | --- |
| **Pubmed** | (((((((((helicobacter pylori[MeSH Terms]) OR (helicobacter pylori[Title/Abstract])) OR (helicobater infections[MeSH Terms])) OR (helicobater infections[Title/Abstract])) OR (HP[Title/Abstract])) OR (H.pylori[Title/Abstract])) OR (h.pylori[Title/Abstract])) OR (helicobater infection[Title/Abstract])) AND ((((((((((probiotics[MeSH Terms]) OR (probiotics[Title/Abstract])) OR (probiotic[Title/Abstract])) OR (bacteria[Title/Abstract])) OR (lactobacillus[Title/Abstract])) OR (bifidobacterium[Title/Abstract])) OR (saccharomyces[Title/Abstract])) OR (lactococcus[Title/Abstract])) OR (bioflor[Title/Abstract])) OR (streptococcus[Title/Abstract]))) AND ((trial[Title/Abstract]) OR (trials[Title/Abstract])) | **307** |
| **EMBASE** | #1 ‘helicobacter pylori'/exp OR 'helicobacter pylori':ab,ti | 74468 |
|  | #2 'h.pylori':ab,ti OR 'hp':ab,ti | 80488 |
|  | #3 'helicobacter infections'/exp OR 'helicobacter infections':ab,ti | 36896 |
|  | #4 'probiotics'/exp OR probiotics:ab,ti | 54384 |
|  | #5 'probiotic'/exp OR probiotic:ab,ti | 55507 |
|  | #6 'bacteria':ab,ti | 528461 |
|  | #7 'lactobacillus':ab,ti | 45852 |
|  | #8 'bifidobacterium':ab,ti | 13988 |
|  | #9 'saccharomyces':ab,ti | 83642 |
|  | #10 'lactococcus':ab,ti | 8399 |
|  | #11 'bioflor':ab,ti | 3 |
|  | #12 'streptococcus':ab,ti | 101420 |
|  | #13 ‘trial’:ab,ti OR ‘trials’:ab,ti | 1822256 |
|  | #14 #1 OR #2 OR #3 | 115314 |
|  | #15 #4 OR #5 OR #6 OR #7 OR #8 OR #9 OR #10 OR #11 OR #12 | 747258 |
|  | **#16 #13 AND #14 AND #15** | **536** |
| **CENTRAL** | #1 MeSH descriptor: [Helicobacter pylori] explode all trees | 2590 |
|  | #2 MeSH descriptor: [Helicobacter Infections] explode all trees | 2492 |
|  | #3 (Helicobacter pylori):ti,ab,kw | 5855 |
|  | #4 (Helicobacter Infections):ti,ab,kw | 2670 |
|  | #5 (H.pylori OR HP):ti,ab,kw | 12908 |
|  | #6 MeSH descriptor: [probiotics] explode all trees | 3004 |
|  | #7 (probiotics):ti,ab,kw | 7107 |
|  | #8 (probiotic):ti,ab,kw | 6762 |
|  | #9 (streptococcus):ti,ab,kw | 5665 |
|  | #10 (bioflor):ti,ab,kw | 5 |
|  | #11 (Lactobacillus):ti,ab,kw | 6191 |
|  | #12 (Bifidobacterium):ti,ab,kw | 3475 |
|  | #13 (Saccharomyces):ti,ab,kw | 683 |
|  | #14 (Lactococcus):ti,ab,kw | 182 |
|  | #15 (bacteria):ti,ab,kw | 16101 |
|  | #16 (trial):ti,ab,kw | 1322890 |
|  | #17 (trials):ti,ab,kw | 2007885 |
|  | #18 #1 OR #2 OR #3 OR #4 OR #5 | 14598 |
|  | #19 #6 OR #7 OR #8 OR #9 OR #10 OR #11 OR #12 OR #13 OR #14 OR #15 | 29076 |
|  | #20 #16 OR #17 | 2008212 |
|  | **#21 #18 AND #19 AND #20** | **802** |

Table S2 PRISMA 2021 Checklist

| **Section and topic** | **Item #** | **Checklist item** | **Location where item is reported** |
| --- | --- | --- | --- |
|
| **Title** | | | |
| Title | 1 | Identify the report as a systematic review. | 1 |
| **Abstract** |  |  |  |
| Abstract | 2 | See the PRISMA 2020 for Abstracts checklist (table 2). | 1 |
| **Introduction** | | | |
| Rationale | 3 | Describe the rationale for the review in the context of existing knowledge. | 2 |
| Objectives | 4 | Provide an explicit statement of the objective(s) or question(s) the review addresses. | 2 |
| **Methods** |  |  |  |
| Eligibility criteria | 5 | Specify the inclusion and exclusion criteria for the review and how studies were grouped for the syntheses. | 2 |
| Information sources | 6 | Specify all databases, registers, websites, organisations, reference lists and other sources searched or consulted to identify studies. Specify the date when each source was last searched or consulted. | 2 |
| Search strategy | 7 | Present the full search strategies for all databases, registers and websites, including any filters and limits used. | 2, Table S1 |
| Selection process | 8 | Specify the methods used to decide whether a study met the inclusion criteria of the review, including how many reviewers screened each record and each report retrieved, whether they worked independently, and if applicable, details of automation tools used in the process. | 2, Figure 1 |
| Data collection process | 9 | Specify the methods used to collect data from reports, including how many reviewers collected data from each report, whether they worked independently, any processes for obtaining or confirming data from study investigators, and if applicable, details of automation tools used in the process. | 2 |
| Data items | 10a | List and define all outcomes for which data were sought. Specify whether all results that were compatible with each outcome domain in each study were sought (e.g. for all measures, time points, analyses), and if not, the methods used to decide which results to collect. | 2 |
|  | 10b | List and define all other variables for which data were sought (e.g. participant and intervention characteristics, funding sources). Describe any assumptions made about any missing or unclear information. | 2 |
| Study risk of bias assessment | 11 | Specify the methods used to assess risk of bias in the included studies, including details of the tool(s) used, how many reviewers assessed each study and whether they worked independently, and if applicable, details of automation tools used in the process. | 2 |
| Effect measures | 12 | Specify for each outcome the effect measure(s) (e.g. risk ratio, mean difference) used in the synthesis or presentation of results. | 2 |
| Synthesis methods | 13a | Describe the processes used to decide which studies were eligible for each synthesis (e.g. tabulating the study intervention characteristics and comparing against the planned groups for each synthesis (item #5)). | 2 |
|  | 13b | Describe any methods required to prepare the data for presentation or synthesis, such as handling of missing summary statistics, or data conversions. | 2 |
|  | 13c | Describe any methods used to tabulate or visually display results of individual studies and syntheses. | 2 |
|  | 13d | Describe any methods used to synthesise results and provide a rationale for the choice(s). If meta-analysis was performed, describe the model(s), method(s) to identify the presence and extent of statistical heterogeneity, and software package(s) used. | 2 |
|  | 13e | Describe any methods used to explore possible causes of heterogeneity among study results (e.g. subgroup analysis, meta- regression). | 2 |
|  | 13f | Describe any sensitivity analyses conducted to assess robustness of the synthesised results. | 2 |
| Reporting bias assessment | 14 | Describe any methods used to assess risk of bias due to missing results in a synthesis (arising from reporting biases). | 2 |
| Certainty assessment | 15 | Describe any methods used to assess certainty (or confidence) in the body of evidence for an outcome. | 2 |
| **Results** |  |  |  |
| Study selection | 16a | Describe the results of the search and selection process, from the number of records identified in the search to the number of studies included in the review, ideally using a flow diagram (see fig 1). | 3, Figure 1 |
|  | 16b | Cite studies that might appear to meet the inclusion criteria, but which were excluded, and explain why they were excluded. | 3, Figure 1 |
| Study characteristics | 17 | Cite each included study and present its characteristics. | 3, Table 1 |
| Risk of bias in studies | 18 | Present assessments of risk of bias for each included study. | Figure S1-2 |
| Results of individual studies | 19 | For all outcomes, present, for each study: (a) summary statistics for each group (where appropriate) and (b) an effect estimate and its precision (e.g. confidence/credible interval), ideally using structured tables or plots. | 4-5 |
| Results of syntheses | 20a | For each synthesis, briefly summarise the characteristics and risk of bias among contributing studies. | 5 |
|  | 20b | Present results of all statistical syntheses conducted. If meta-analysis was done, present for each the summary estimate and its precision (e.g. confidence/credible interval) and measures of statistical heterogeneity. If comparing groups, describe the direction of the effect. | 5 |
|  | 20c | Present results of all investigations of possible causes of heterogeneity among study results. | 5 |
|  | 20d | Present results of all sensitivity analyses conducted to assess the robustness of the synthesised results. | 5 |
| Reporting biases | 21 | Present assessments of risk of bias due to missing results (arising from reporting biases) for each synthesis assessed. | 5 |
| Certainty of evidence | 22 | Present assessments of certainty (or confidence) in the body of evidence for each outcome assessed. | 5 |
| **Discussion** |  |  |  |
| Discussion | 23a | Provide a general interpretation of the results in the context of other evidence. | 9-10 |
|  | 23b | Discuss any limitations of the evidence included in the review. | 9-10 |
|  | 23c | Discuss any limitations of the review processes used. | 9-10 |
|  | 23d | Discuss implications of the results for practice, policy, and future research. | 9-10 |
| **Other information** |  |  |  |
| Registration and protocol | 24a | Provide registration information for the review, including register name and registration number, or state that the review was not registered. | 10 |
|  | 24b | Indicate where the review protocol can be accessed, or state that a protocol was not prepared. | 10 |
|  | 24c | Describe and explain any amendments to information provided at registration or in the protocol. | 10 |
| Support | 25 | Describe sources of financial or non-financial support for the review, and the role of the funders or sponsors in the review. | 10 |
| Competing interests | 26 | Declare any competing interests of review authors. | 10 |
| Availability of data, code, and other materials | 27 | Report which of the following are publicly available and where they can be found: template data collection forms; data extracted from included studies; data used for all analyses; analytic code; any other materials used in the review. | 10-11 |

From: EA, Brennan SE, Chou R, Glanville J, Grimshaw JM, Hróbjartsson A, Lalu MM, Li T, Loder EW, Mayo-Wilson E, McDonald S, McGuinness LA, Stewart LA, Thomas J, Tricco AC, Welch VA, Whiting P, Moher D. The PRISMA 2020 statement: an updated guideline for reporting systematic reviews. BMJ.2021;372:n71.

Table S3 Characteristics of studies included in the meta analysis

| First author, year | Region | Study design | No. of patients included(exp/cont.) | Probiotic supplement | Test for confirming eradication | | regimens compared |
| --- | --- | --- | --- | --- | --- | --- | --- |
|  |  |  |  |  | Time(w) | Test |  |
| Armuzzi, 2001 | Italy | RCT-2 arm | 60(30/30) | Lac | 6 | ^13^C-UBT | Triple therapy with probiotic vs Triple therapy |
| Canducci, 2000 | Italy | RCT-2 arm | 120(60/60) | Lac | 6 | ^13^C-UBT/ histology | Triple therapy with probiotic vs Triple therapy |
| Deguchi, 2011 | Japan | RCT-2 arm | 229(115/114) | Lac | 8 | UTB/stool antigen test | Triple therapy with probiotic vs Triple therapy |
| Duman, 2005 | Turkey | RCT-2 arm | 389(204/185) | Sac | 6 | NA | Triple therapy with probiotic vs Triple therapy |
| Emara, 2014 | Egypt | RCT-2 arm | 70(35/35) | Lac | 4 | microscopical examination/ histology/stool antigen test | Triple therapy with probiotic vs Triple therapy |
| Francavilla, 2013 | United States | RCT-2 arm | 100(50/50) | Lac | 8 | ^13^C-UBT | Triple therapy with probiotic vs Triple therapy |
| Grgov, 2016 | Serbia | RCT-2 arm | 167（90/77） | Bif+Lac+Sac | 8 | microscopical examination/histology/RUT | Triple therapy with probiotic vs Triple therapy |
| Haghdoost, 2017 | Iran | RCT-2 arm | 176(88/88) | Bif+Lac | 6 | stool antigen test | Triple therapy with probiotic vs Triple therapy |
| Hauser, 2014 | Croatia | RCT-2 arm | 804（398/406） | Bif+Lac | 6 | UBT/rapid urease test/stool antigen | Triple therapy with probiotic vs Triple therapy with placebo |
| Lee, 2011 | Korea | RCT-2 arm | 223(107/116) | Sac | 4 | ^13^C-UBT | Triple therapy with probiotic vs Triple therapy |
| Medeiros, 2011 | Portugal | RCT-2 arm | 62(31/31) | Lac | 6 | ^13^C-UBT | Triple therapy with probiotic vs Triple therapy |
| Mihai, 2019 | Romania | RCT-2 arm | 70(35/35) | Lac | 6 | RUT | Triple therapy with probiotic vs Triple therapy |
| Muresan,2019 | Romania | RCT-2 arm | 46(23/23) | Lac | 4 | stool antigen test | Triple therapy with probiotic vs Triple therapy |
| Myllyluoma, 2005 | Finland | RCT-2 arm | 47(23/24) | Lac+Pro | 16 | ^13^C-UBT/serology test | Triple therapy with probiotic vs Triple therapy |
| Nista, 2004 | Italy | RCT-2 arm | 104(53/51) | Bac | 6 | ^13^C-UBT | Triple therapy with probiotic vs Triple therapy with placebo |
| Ojetti, 2012 | Italy | RCT-2 arm | 90(45/45) | Lac | 6 | ^13^C-UBT | Triple therapy with probiotic vs Triple therapy |
| Paoluzi, 2015 | Italy | RCT-2 arm | 142(71/71) | Lac | 8 | UTB/stool antigen test | Triple therapy with probiotic vs Triple therapy |
| Sheu, 2002 | Taiwan | RCT-2 arm | 160(80/80) | Bif+Lac | 8 | ^13^C-UBT/histology/RUT/ | Triple therapy with probiotic vs Triple therapy |
| Shimbo, 2005 | Japan | RCT-2 arm | 35(17/18) | Bac | 6 | microscopical examination/RUT/culture method | Triple therapy with probiotic vs Triple therapy |
| Sung, 2008 | Greece | RCT-2 arm | 352(176/176) | Bac+Str | 4 | NA | Triple therapy with probiotic vs Triple therapy |
| Tongtawee,2015 | Thailand | RCT-2 arm | 200(100/100) | Lac+Str | 4 | histology/RUT | Triple therapy with probiotic vs Triple therapy |
| Yang, 2021 | China | RCT-2 arm | 200(100/100) | Lac | 8 | ^13^C-UBT | Triple therapy with probiotic vs Triple therapy |
| Yoon, 2011 | Korea | RCT-2 arm | 337(151/186) | Bif+Lac+Str | 4 | ^13^C-UBT/histology/RUT | Triple therapy with probiotic vs Triple therapy |
| Zojaji, 2013 | Iran | RCT-2 arm | 160(80/80) | Sac | 8 | ^13^C-UBT | Triple therapy with probiotic vs Triple therapy |
| D'Angelo, 2014 | Italy | RCT-3 arm | 300(100/100/100) | Bac | 4 | UBT | Sequential therapy vs Triple therapy with probiotic vs Triple therapy |
| Chang, 2020 | Korea | RCT-3 arm | 183(61/61/61) | Sac | 4 | ^13^C-UBT | Triple therapy with probiotic vs Triple therapy with sulforaphane vs Triple therapy |
| Dajani, 2012 | United Arab Emirates | RCT-3 arm | 377(100/95/106) | Bac | 6-8 | ^14^C-UBT | Triple therapy with probiotic added at the same time vs starting the probiotic for 2 weeks before Triple therapy vs Triple therapy |
| Du, 2012 | China | RCT-3 arm | 234(79/78/77) | Lac | 4 | ^13^C-UBT | Triple therapy with probiotic before vs Triple therapy with probiotic after vs Triple therapy |
| Ozdil, 2011 | Turkey | RCT-3 arm | 285（98/95/92) | Sac | 5 | stool antigen test | Triple therapy with probiotic vs Triple therapy vs Sequential therapy |
| Song, 2010 | Korea | RCT-3 arm | 991(331/330/330) | Sac | 4 | ^13^C-UBT | Triple therapy with probiotic vs Triple therapy with probiotic and mucoprotective agent vs Triple therapy |
| Ziemniak, 2006 | Poland | RCT-3 arm | 641(53/192/241) | Lac | NA | ^13^C-UBT | Triple therapy with probiotic vs Triple therapy vs Quadruple therapy |
| Cremonini, 2004 | Italy | RCT-4 arm | 85(21/22/21/21) | Bif+Lac+Sac | 5-7 | ^13^C-UBT | Triple therapy with Lactobacillus GG vs Triple therapy with Saccharomyces boulardii vs Triple therapy with Lactobacillus spp. and biphidobacteria vs Triple therapy with placebo |
| Scaccianoce, 2008 | Italy | RCT-4 arm | 65(17/15/17/16) | Bif+Lac+Str | 4-6 | ^13^C-UBT | Triple therapy 7d with probiotic vs Triple therapy 7d with probiotic mixture vs Triple therapy 14d with probiotic mixture vs Triple therapy 7d |
| Kim, 2019 | Korea | RCT-5 arm | 1500(300/300/300/300/300) | Lac | 6 | ^13^C-UBT | Triple therapy with probiotic vs Triple therapy vs sequential therapy vs concomitant therapy vs tailored therapy |

Table S4 Result of subgroup analyses for side effect

| Subgroup | No. of arms | Sample size | RR(95% CI) | P_effect_ | I^2^ (%) | P_heterogeneity_ |
| --- | --- | --- | --- | --- | --- | --- |
| PPI type |  |  |  |  |  |  |
| Lansoprazole | 8 | 1094 | -0.19 [-0.33, -0.04] | 0.01 | 90 | <0.01 |
| Esomeprazole | 7 | 920 | -0.10 [-0.21, 0.00] | 0.05 | 71 | <0.01 |
| Rabeprazole | 6 | 323 | -0.26 [-0.55, 0.03] | 0.08 | 93 | <0.01 |
| Omeprazole | 4 | 1352 | -0.10 [-0.16, -0.03] | <0.01 | 62 | 0.05 |
| Pantoprazole | 3 | 358 | -0.06 [-0.13, 0.02] | 0.38 | 0 | 0.13 |
| Unclear | 4 | 1311 | -0.23 [-0.28, -0.18] | <0.01 | 0 | 0.76 |
| Antibiotic type |  |  |  |  |  |  |
| Amoxicillin+Clarithromycin | 22 | 3793 | -0.14 [-0.20, -0.09] | <0.01 | 79 | <0.01 |
| Tinidazole+Clarithromycin | 4 | 159 | -0.38 [-0.51, -0.24] | <0.01 | 15 | 0.32 |
| Levofloxacin+Doxycycline | 2 | 103 | -0.16 [-0.65, 0.33] | 0.52 | 85 | <0.01 |
| Amoxicillin+Moxifloxacin | 1 | 337 | 0.03 [-0.06, 0.13] | 0.51 | NA | NA |
| Unclear | 2 | 904 | -0.21 [-0.27, -0.15] | 0.92 | 0 | <0.01 |
| Publication year |  |  |  |  |  |  |
| 2000-2011 | 18 | 2650 | 0.62 [0.48, 0.79] | <0.01 | 64 | <0.01 |
| 2012-2022 | 14 | 2708 | 0.60 [0.49, 0.73] | <0.01 | 73 | <0.01 |
| Triple therapy duration |  |  |  |  |  |  |
| 7d | 17 | 1967 | -0.18 [-0.28, -0.09] | <0.01 | 84 | <0.01 |
| 10d | 4 | 653 | -0.17 [-0.30, -0.05] | <0.01 | 81 | <0.01 |
| 14d | 9 | 1734 | -0.10 [-0.17, -0.03] | <0.01 | 76 | <0.01 |
| Unclear | 1 | 804 | -0.21 [-0.27, -0.15] | NA | NA | <0.01 |
| Follow-up time |  |  |  |  |  |  |
| 0-4w | 12 | 1841 | 0.64 [0.51, 0.81] | 0.02 | 52 | <0.01 |
| 4-8w | 18 | 3133 | 0.51 [0.41, 0.64] | <0.01 | 76 | <0.01 |
| >8w | 3 | 323 | 0.82 [0.59, 1.15] | 0.25 | 27 | 0.25 |
| Unclear | 1 | 200 | 0.95 [0.77, 1.18] | NA | NA | 0.66 |
| Location |  |  |  |  |  |  |
| Europe and America | 19 | 2211 | 0.68 [0.63, 0.74] | 0.04 | 42 | <0.01 |
| Eastern Europe | 6 | 1202 | 0.38 [0.30, 0.49] | 0.35 | 10 | <0.01 |
| Asia | 9 | 2167 | 0.74 [0.65, 0.84] | <0.01 | 76 | <0.01 |
| Regimen duration |  |  |  |  |  |  |
| <7d | 10 | 772 | 0.55 [0.38, 0.79] | <0.01 | 70 | <0.01 |
| 7-14d | 11 | 1791 | 0.54 [0.38, 0.77] | <0.01 | 79 | <0.01 |
| >14d | 8 | 1487 | 0.61 [0.44, 0.83] | 0.01 | 64 | <0.01 |
| Unclear | 3 | 1308 | 0.79 [0.57, 1.11] | 0.04 | 70 | 0.48 |
| Regimen adding time |  |  |  |  |  |  |
| Before triple therapy | 4 | 553 | 0.67 [0.43, 1.04] | <0.01 | 88 | 0.08 |
| After triple therapy | 14 | 1476 | 0.60 [0.46, 0.78] | <0.01 | 57 | <0.01 |
| With triple therapy | 1 | 42 | 0.23 [0.08, 0.69] | NA | NA | <0.01 |
| Longer than triple therapy | 11 | 2041 | 0.53 [0.41, 0.70] | 0.01 | 56 | <0.01 |
| Unclear | 3 | 1308 | 0.79 [0.57, 1.11] | 0.04 | 70 | 0.48 |
